# Performance evaluation of an operational dengue forecasting system (D-MOSS) in Vietnam

**DOI:** 10.1371/journal.pgph.0005867

**Published:** 2026-03-06

**Authors:** Amy Marie Campbell, Felipe Colón-González, Do Kien Quoc, Nguyen Hai Tuan, Nguyen Thanh Dong, Tran Thi Trang, Lokman Hakim Bin Sulaiman, Shew Fung Wong, Barbara Hofmann, Gina Tsarouchi, Quillon Harpham, Vu Sinh Nam, Oliver Brady

**Affiliations:** 1 London School of Hygiene and Tropical Medicine, London, United Kingdom; 2 Wellcome Trust, London, United Kingdom; 3 Pasteur Institute, Ho Chi Minh City, Vietnam; 4 National Institute of Hygiene and Epidemiology, Hanoi, Vietnam; 5 Pasteur Institute, Nha Trang, Vietnam; 6 Tay Nguyen Institute of Hygiene and Epidemiology, Dak Lak, Vietnam; 7 IMU University, Kuala Lumpur, Malaysia; 8 HR Wallingford, Oxfordshire, United Kingdom; Mahidol University, THAILAND

## Abstract

D-MOSS (Dengue forecasting Model Satellite-based System) was launched operationally in Vietnam in June 2019, providing near-real time dengue forecasts across all 63 provinces. Very few dengue forecasting systems have prospectively evaluated the performance of dengue forecasting under real-world operational conditions. This study comprehensively assesses D-MOSS dengue forecasting performance since operationalisation through both statistical accuracy (absolute dengue incidence, trajectory of incidence, timing of peaks), and operational utility (predictions for specific decision-making scenarios). The D-MOSS dengue forecasts in Vietnam outperformed null model baselines across almost all performance metrics.. While lead times of one month reported the highest accuracy, there was no steep linear decline in accuracy as lead times increased up to six months, and the greatest value-added over seasonal average baseline models was found for later lead times at four to six months. Higher value-added differences were observed for the second half of the year, but the unusually-early June dengue outbreak in 2022 provided a notable challenge. Spatially, larger errors were found in central and southern provinces, that report higher dengue incidence, alongside contrastingly greater value-added over null baseline models, particularly at shorter lead times. Four contextualised operational utility scenarios were tested through probabilistic classification of outbreak threshold exceedance, with accuracy ranging across probability cut-offs but peaking at 0.83 - 0.94 across scenarios.The value of waiting for the next month’s forecast and utilising different outbreak thresholds was assessed, with heterogeneous results and the distribution of false alarms or missed outbreaks clustering spatially. The results of both the global performance analysis and utility assessments continue to highlight the strong predictive ability of D-MOSS in an operational setting. Lessons should be taken from the higher-than-expected performance over long-term horizons to improve our ability to forecast further into the future, amidst the key insights these results provide into the improvement of operational dengue forecasting for key decision-making situations in Vietnam and beyond.

## Introduction

Dengue fever is a vector-borne disease caused by four viral serotypes (DENV-1–4), and transmitted by infected *Aedes* mosquito bites in tropical and subtropical climates across Asia, the Americas and Africa [1,2]. Most dengue fever infections are self-limiting or present asymptomatically, however less frequent severe dengue results in haemorrhagic fever and dengue shock syndrome which can be life-threatening, usually associated with reinfection [3]. A predicted 5.69 billion people, 73% of global population, live in areas suitable for dengue transmission [4], with dengue incidence more than doubling in recent decades in regions including Southeast Asia [5].

Due to current limited uptake of the live dengue vaccine [6,7] and a lack of specific antiviral treatment for dengue [8], transmission reduction and interventions centre around vector control, such as insecticide-based methods to reduce mosquito populations. Vector populations themselves have a range of seasonal and spatial drivers, meaning dengue dynamics function within a complex interface of human, environment and mosquito dynamics. Efforts to quantify and model such relationships have increased our capabilities to predict dengue dynamics, ranging from mosquito habitat suitability [9] to dengue outbreak forecasting models [10]. Accurate and timely forecasting of dengue outbreaks and trends can limit transmission in a decision-support capacity, triggering anticipatory interventions to limit the impact on human health and healthcare resources [11]. Resources and interventions can be targeted proactively and specifically, preceding the onset of reported cases, within target areas and time-windows, which can combat rising insecticide resistance in *Aedes* vectors [12]. Most dengue forecasting models are research-focused and only assessed retrospectively. Operational deployment with real-time refitting is accompanied by challenges around data flows and non-ideal real-world conditions, so operational evaluation is crucial to understand performance in practice and determine whether forecasts can reliably support interventions, quantifying the efficacy to effectiveness gap for infectious disease forecasting.

Vietnam reported an average 130,235 dengue cases a year between 2013–2019 according to the OpenDengue database [13,14], which causes a significant health burden. These cases are mostly associated with *Aedes aegypti* populations and occasionally *Aedes albopictus* [15], in urban and peri-urban areas [16]. As in all dengue-endemic countries, this number is likely underestimated due to mild or asymptomatic infections and non-specific febrile symptoms [17]. Previously, monitoring of dengue dynamics relied on outbreak alert thresholds related to the Vietnamese government’s Decision No. 02/2016/QD-TTg (with outbreaks declared when the number of cases in a commune exceeds the monthly average of the same period over the past three years).

The D-MOSS (Dengue forecasting Model Satellite-based System) forecasting system aimed to produce more effective early-warning forecasts of dengue incidence, developed under the guidance of local stakeholders, including the Ministry of Health of Vietnam, WHO regional office, the Nha Trang and Ho Chi Minh Pasteur Institutes, province-level health authorities, the Vietnamese National Institute of Hygiene and Epidemiology, and the Tay Nguyen Institute of Hygiene and Epidemiology, through engagement workshops, meetings and surveys. The D-MOSS forecasting system was launched operationally in Vietnam in June 2019, providing near-real time dengue forecasts across the 63 provinces, representing the first prospective, routine dengue forecasting system based on Earth Observation data in operational use. The Vietnam D-MOSS forecasting system uses a superensemble of 5 probabilistic spatiotemporal hierarchical dengue models, using prospective lagged dengue cases, 42 seasonal climate forecast ensembles from the UK Met Office Global Seasonal Forecasting System version 5 (GloSea5) [18] and further Earth Observation data sources as climatic and sociodemographic predictors. The full statistical model design at the core of the D-MOSS implementation in Vietnam, and associated accuracy assessment based on historical data, is detailed in Colón-González et al. [17]. D-MOSS generates near-real time probabilistic forecasts of dengue cases at a monthly frequency and province-level (administration level 1) resolution, across a forecast horizon with lead times of 1–6 months. The model is refitted on a monthly frequency, appending the latest dengue case data and covariate data and recalculating the coefficients prior to running the model [19]. Alongside monthly point estimates of dengue cases and incidence (per 100,000 people) for each province, D-MOSS provides a probability of a predefined dengue outbreak threshold being exceeded. Users are able to select between four outbreak thresholds; two using percentiles (75^th^ and 95^th^) or two using deviation above the mean (1 or 2 standard deviations), based on an endemic channel baseline [17]- a moving average representative of the usual seasonal range of cases, as per other dengue control decision-making procedures [20].

Placing D-MOSS in the greater context of dengue forecasting, existing and alternative dengue forecasting systems can be categorised into multiple levels. The first level of dengue forecasting involves detecting significant case increases and predicting whether this might lead to outbreaks, such as the Early Warning And Response System (EWARS) framework [21,22], which has been assessed for dengue forecasting in Mexico [20,23], Colombia [24], Brazil [20] and Malaysia [20], with performance ranging widely in terms of positive predictive values (40–88%) and sensitivity (57–97%). The second level uses current, real-time or historical climate data to predict future dengue cases based on lagged associations with climate variables. However, these early warning systems are triggered based on observable signals, such as an initial increase in cases [25], which can only provide short notice of an outbreak, which might be insufficient for adequate response. The third level of dengue forecasting involves prospectively predicting future conditions based on seasonal climate forecasts. Here, the predictions are made well before signals can be observed in current climate or surveillance data that usually trigger early warning systems, as the signals themselves are being forecasted at horizons of up to 6 months ahead, as in the D-MOSS dengue forecasting system, facilitating preventative action at an earlier stage. Compared to other dengue forecasting systems designed for priority areas, D-MOSS is a nationally operational model in a country where dengue dynamics exhibit a range of seasonal, interannual and spatial variability- with notably lower and more sporadic incidence in northern Vietnam, and more endemic incidence in central and southern Vietnam with large interannual variation [26], ranging from large population centres to rural areas.

These inherent design differences necessitate an evaluation of the specific performance possible for this distinct operational dengue forecasting system, going beyond binary classifications of outbreak alarm accuracy, by comprehensively assessing performance across a range of forecast outcomes that encompass dengue dynamics and patterns and assessing utility in the context of specific decision-making scenarios in an operational context. While the model performance has been evaluated based on hindcast dengue forecasts on historical data [17], the real-time performance of its prospective dengue forecasts since operationalisation, under real-world has not yet been fully evaluated. This can provide valuable insights into the system’s adaptability, identify areas where further refinement is required, and provide evidence to continue strengthening confidence among authorities to support continued and long-term integration into dengue control.

The key aims were therefore to comprehensively assess D-MOSS dengue forecasting performance since operationalisation through both statistical accuracy and operational utility. D-MOSS performance was evaluated:

1. Compared to baseline models representing previous dengue monitoring approaches, to demonstrate predictive value added by a dengue forecasting system;
2. Across the full range of lead times to explore forecast horizon accuracy;
3. Across spatial and seasonal dimensions to highlight spatiotemporal variations in performance;
4. For key decision-making scenarios.

This assessment can provide recommendations for future areas of development and improvement to the D-MOSS system, and guidance for how decision makers in different settings can most effectively integrate dengue forecasts from the DMOSS system into dengue management protocols.

## Methods

### Operational adjustments to D-MOSS model implementation

Since the publication of the model implementation in Colón-González et al. [17], minor adjustments have been made to the model for its operational deployment.

Five additional climate covariates were added; mean temperature, potential and actual evapotranspiration, total soil moisture content and runoff. Actual evapotranspiration (ETₐ), potential evapotranspiration, runoff, and soil moisture were simulated using the Variable Infiltration Capacity (VIC) model [27]. The VIC model was run in full energy balance mode at a 6-hour timestep, driven by meteorological forcing from GloSea [18] (precipitation, air temperature, wind speed, radiation, and vapour pressure), together with land surface parameters including soil properties (texture, hydraulic conductivity, layer depths) and vegetation characteristics (leaf area index, root depth, and fractional cover), defined on a 0.25° × 0.25° grid. Forecasts were produced for the full GloSea [18] ensemble set (typically 42 members) over a 6-month horizon. ETₐ was estimated using a water balance approach that accounts for soil moisture availability and potential evaporation. Potential evapotranspiration was calculated as the area-weighted sum of potential transpiration and soil evaporation, based on vegetation characteristics and atmospheric conditions, assuming no environmental limitations. Runoff was generated by partitioning precipitation into surface and subsurface components, using a variable infiltration curve to represent sub-grid variability in soil properties. Soil moisture was simulated for each of the model’s soil layers (typically three), with total moisture content defined as the sum of liquid water and ice in each layer. The addition of the hydrological model outputs increased predictability in the models during covariate selection, and also resulted in a previous meteorological variable (mean temperature) becoming predictive, leading to its inclusion.

The superensemble model structure was adjusted, retaining the top four highest performing models in terms of predictability, and including an additional baseline model set-up. Instead of Bayesian Model Averaging to combine the models, which was complex and produced almost identical weights for all models, the 4 best models were selected and combined based on weights generated from the CRPS of 1000 samples generated from the posterior marginal distribution of each model’s predictions against observations for historical data, which are recalculated monthly alongside the updating of covariates and refitting. The normalized weights are given by:


$$\tilde{w_{s}}=\frac{\frac{\text{1}}{w_{s}^{\text{2}}}}{\sum_{i=\text{1}}^{n}\frac{\text{1}}{w_{s,i}^{\text{2}}}}$$

where $\tilde{w_{s}}$ is the normalised weight for each model (*s*), $w_{s}$ is the CRPS value for each model (*s*), and *n* is the total number of models. This provided more interpretable and heterogenous results than the Bayesian Model Averaging approach to select and weight models. Finally, additional output variables were generated, including the length of the dengue transmission season (based on months when incidence exceeded 10 cases per 100,000), and the proportion of variation explained by the predictors, based on recommendations from stakeholder engagement.

### Experimental design for performance evaluation

Historical dengue forecasts generated by D-MOSS in Vietnam during the operational period July 2019 to September 2022 were evaluated against observed monthly dengue incidence for the same operational period and for all 63 provinces in Vietnam.

The analysis was split into two approaches; the first was the acquisition of global performance metrics assessing the accuracy of point-estimates of dengue incidence across a range of spatiotemporal dimensions, including a comparison against a randomly-sampled per-province baseline, and seasonally expanding average per-province baseline, to demonstrate predictive value added by a dengue forecasting system. The second approach selected specific decision-making situations from which to assess the quality of information available within operational scenarios, following a more contextualised utility assessment approach and based on probabilistic accuracy of outbreak thresholds being exceeded.

### D-MOSS forecast outcome variables

The D-MOSS forecasting system produces a range of dengue forecast outcome variables including cases, incidence, outbreak exceedance probabilities, and variance explained by predictors. The population-weighted dengue incidence rate per 100,000 people was selected as the outcome variable for the global performance analysis, in the form of point-estimates. These point-estimate incidence rates are calculated within the model implementation, taking an ensemble mean of predicted dengue cases across the means of the posterior predictive distribution of all 4 models in the superensemble for each month and province, and normalising to a rate per 100,000 people using province-level population data from Gridded Population of the World (GPW) data [28]. As the superensemble model was run on 42 seasonal climate forecast ensemble members, generating 42 predictions for each province and month, the arithmetic mean across the 42 seasonal climate forecast was obtained for a single incidence forecast value per province and month. The rationale for choosing incidence rate rather than the raw number of cases was to facilitate effective comparison across Vietnam, without high population areas skewing trends of accuracy. The outbreak exceedance probabilities provided to users were not used as an outcome variable in these initial global performance assessments, as the outbreak definitions differ regionally across the country due to heterogenous local dengue management, and change dynamically over space and time which has implications for timeliness of interventions [29]. While the ensemble mean of incidence overlooked the probabilistic capabilities of the D-MOSS superensemble, it represents an output provided to users that is uniform across all regions, regardless of specific operational context.

Both these elements- the probabilistic nature of the superensemble, and the outbreak exceedance outputs- were instead assessed under the operational utility scenarios in the second part of the analysis. The setting of outbreak thresholds is relative based on specific operational context, so it was more relevant to explore the accuracy of the probabilities of exceeding these in relation to specific scenarios. This also ensured full coverage of the range of outcome variables (both point-based and probabilistic) provided to users by the D-MOSS forecasting system in this performance assessment.

### Performance metric calculation

Incidence data acquired for both Vietnam D-MOSS dengue forecasts and observed dengue cases over the operational period were merged into a single dataframe, based on province ID (n = 63) and timestamp (n = 39 months). For each unique combination of province and month, there was a single observed value of dengue incidence, alongside the D-MOSS forecasted values of dengue incidence for that month generated at lead times of 1–6 months prior.

To assess the global performance of the D-MOSS dengue forecasting system in Vietnam, we evaluated metrics that captured different indicators of dengue forecasting performance. We chose 3 specific outcomes that summarise the key dengue dynamics an effective dengue forecasting model would predict, including patterns and peaks rather than just focusing on raw incidence alone, to consider the wider needs of decision-makers, and selected metrics that measured the accuracy of these specific outcomes.

Firstly, the root mean squared error (RMSE) of incidence (cases per 100,000 people) between the observed and forecasted values were calculated in R using scoringutils v2.0.0 [30]. RMSE is a widely-used and standard metric to measure the magnitude of errors between observed and forecasted continuous values, with the square root of the value providing metrics in the same units as the response variable (dengue incidence), which increases interpretability to allow public health officials to quantify the differences between observed and D-MOSS forecast incidence rates.

Secondly, the temporal difference between when dengue cases were observed to peak, and when D-MOSS forecasts predicted cases peaking, was calculated in months, based on the maximum incidence rate each calendar year, using data processing packages dplyr v1.1.4 [31] and tidyr v1.3.1 [32] in R. This simple and direct metric captures the accuracy of the forecast at an epidemiologically-relevant timepoint, in terms of when an outbreak is most severe and will represent the greatest burden, which can support outbreak management such as effectively mobilizing resources to manage outbreak peaks- which is particularly helpful for planning in regions with strong dengue seasonality.

Thirdly, the trajectory accuracy of dengue accuracy was assessed through a distance metric calculated by dynamic time warping (DTW), using dtw v1.23-1 [33] in R. This distance metric represents the minimal cumulative cost of aligning the observed time series of dengue cases with D-MOSS forecasted time series of dengue cases:


$$DTW\;(A,\;B)=\text{min}\sqrt{\sum_{i,j}{d\left(a_{i},\;b_{j}\right)}^{\text{2}}}$$

where $d\left(a_{i},\;b_{j}\right)$ is the distance between points $a_{i}$ from the observed time series and $b_{j}$ from the forecasted time series, and the sum is taken across all points in the optimally aligned (minimum cost) path. DTW was conducted separately for each lead time to allow comparison of forecast pattern accuracy across lead times. Instead of a simple Euclidean distance between point-estimates of incidence, this metric can provide insights for trajectories that can be similarly shaped but misaligned temporally, to evaluate D-MOSS performance in capturing the pattern and progression of outbreaks, rather than solely point-wise accuracy. Vietnam experiences patterns of low dengue incidence in the dry season, and higher incidence in the rainy season. Accurate pattern forecasting facilitates long-term planning in response to the direction or gradient of incidence trends, including assessing the impact of interventions, even if the individual point values themselves are incorrect.

The distribution of each of these observed outcomes were first explored in the observed dengue cases, e.g., spatiotemporal patterns of incidence, seasonality of incidence and when dengue cases tended to peak in each province. Each of these metrics were then generated overall to quantify how accurately these observed outcomes were replicated in the corresponding D-MOSS dengue forecasts for Vietnam, encompassing all seasonal climate forecast ensembles and lead times unless otherwise stated. These chosen metrics were then generated across a variety of dimensions; along the lead times (forecast horizon), across months (temporal variation) and across provinces (spatial variation).

### Metric comparison with baseline approaches

To assess the dengue forecasting performance increase added by the D-MOSS forecasting system in Vietnam, compared to non-modelling approaches, the metrics generated were compared to those metrics generated for a random baseline and simpler seasonal approach. Firstly, a random baseline was generated by simulating dengue ‘forecasts’ through random sampling of the observed dengue incidence values per-province, for the operational period,. Secondly, a seasonal expanding average baseline was created per-province using observed dengue cases, to represent a basic non-modelling baseline that accounted for seasonality, and to facilitate assessment on the value-added from the more complex D-MOSS modelling approach using the superensemble and its full range of covariates as described in Colón-González et al. [17]. This seasonal expanding average adds more context than a random predictor, by capturing seasonality in dengue incidence, while gradually adjusting to any longer-term shifts or patterns, mimicking the behaviour of D-MOSS monthly refitting protocols. It was computed using observed dengue cases data from August 2002, for the full operational period, using a cumulative mean for each calendar month based on calendar months in previous years to progressively refine the prediction.

Performance metrics as described previously were generated for both of these baseline approaches with the same methodologies to allow accurate comparison through difference subtraction, to quantify value-added by D-MOSS. Alongside these relative comparisons, the absolute performance metrics were also reported, with both expected to provide insights into D-MOSS performance. For example, in areas where a seasonal baseline model might function more effectively, such as endemic areas in the south, a lower relative improvement could be reported but this would not necessarily be indicative of a poorer forecast, requiring both absolute and relative metrics to be assessed in combination.

### Operational scenario utility assessment

Four key operational scenarios were selected, based on previous engagement with D-MOSS users, encompassing different decision-making protocols. These were co-developed over a series of meetings and workshops with representatives of the Vietnamese Ministry of Health across the country, from local to national levels and spanning multiple sectors (mosquito control, epidemiology, meteorology, budgeting and planning), between March 2018 and May 2019. These meetings included hypothetical outbreak management “wargame” exercises where potential use cases at different stages of an outbreak in their local settings were discussed. The resulting scenarios are used here to assess utility performance, consisting of: budget allocation, forecasting based on weather conditions, early warning of cases progressing into outbreaks, and outbreak management (Table 1). For each of these scenarios, particular subsets of the observed and forecasted data were selected to represent the scenario (S1 Fig), for each unique province or outbreak period in the year 2020. The rationale for selecting this year was twofold. Firstly, 2020 was chosen due to its completeness as a full year of data within the operational period (unlike 2019 and 2022). Secondly, the annual incidence in 2020 (138,693 cases) lies significantly closer to the average number of cases over 2013–2019 of 130,235 dengue cases a year, based on the OpenDengue database (Clarke et al., 2023; Clarke et al., 2024), compared to the low incidence reported in 2021 (69,335 cases). Possible consequences of choosing this year for utility assessments are discussed below.

**Table 1:** Utility accuracy assessment criteria. All scenarios tested dengue forecasts issued in April and May 2020 for a forecast horizon of June to October. Observed events were classified into a binary variable which was assessed against the probabilistic forecasts produced by D-MOSS, in terms of exceedance probabilities of outbreak thresholds. While analysis was conducted for all four outbreak thresholds within the D-MOSS implementation (mean plus one standard deviation, mean plus two standard deviation, the 75^th^ quantile, and the 95th quantile), hereafter results focus on the mean plus two standard deviation outbreak threshold.

Operational scenario	Operational question	Selection criteria	Probabilistic Classification
1. Budget Allocation	Will this be an above-average dengue year?	All provinces included.	Observed: Binary variable of whether that province had more months exceeding the selected outbreak threshold than usual (1, n = 8), based on average from 2002-2022, or not (0, n = 55). Forecasted: Mean of forecast probabilities of each month in forecast horizon exceeding the selected threshold produced by D-MOSS.
2. Forecasting	Will this weather lead to more dengue cases than average?	Selected provinces where March-May mean precipitation exceeded average (based on 2002–2022).	Observed: Binary variable of whether the province exceeded the selected outbreak threshold that month (1, n = 18), or not (0, n = 267). Forecasted: Probability of that province exceeding the selected outbreak threshold that month, produced by D-MOSS.
3. Early warning	Will these dengue cases lead to an outbreak?	Selected provinces with consecutive increases in dengue incidence between months March-May.	Observed: Binary variable of whether the province exceeded the selected outbreak threshold that month (1, n = 9), or not (0, n = 41). Forecasted: Probability of that province exceeding the selected outbreak threshold that month, produced by D-MOSS.
4. Outbreak management	When will this dengue outbreak peak?	Selected provinces that exhibited ‘outbreak events’; defined as 3 consecutive months exceeding the selected outbreak exceedance threshold between June and October.	Observed: Binary variable highlighting which month had the highest exceedance of the threshold in that province (1, n = 8), or not (0, n = 32). Forecasted: Probability of that province exceeding the selected outbreak threshold that month, produced by D-MOSS.

All assessments focused on a forecast horizon period from June to October (Table 1, S1 Fig), which generally contains the majority of reported dengue cases in Vietnam (61.2%), comparing information available at the point of the April forecast (the first decision-making opportunity month, lead times 2–6) compared to the difference in information value available one month later in the May forecast (the second decision-making opportunity month, lead times 1–5). While generally annual budgets are set at the start of the fiscal year, the allocation of these funds towards intervention programmes likely happens later in the year, at the start of the dengue season, so dengue forecasts made in April and May can be pivotal for timely and focused dengue response.

All provinces were used for the ‘budget allocation’ scenario, however the other scenarios underwent a selection criteria to quantitatively select particular conditions that fulfilled the scenario criteria (Table 1). To explore decision-making as a response to anomalous weather conditions within the ‘forecasting’ scenario, provinces were selected where precipitation in the pre-dengue season (March to May) that year exceeded the per-province average precipitation (a 3-month average based on 2002–2022). Increased precipitation has been found to significantly increase dengue cases across provinces in Vietnam at lag times of 0–3 months [34], associated with increasing potential mosquito breeding sites. The months of March to May in Vietnam mark a transitional period out of the dry season, a period that influences mosquito breeding site formation that underpins later dengue transmission. This ‘forecasting’ scenario thus aimed to capture how effectively this climactic predictor’s impact on later dengue outbreaks could be assessed for operational decision-making based on current weather conditions. Similarly, within the ‘early warning’ scenario, provinces where dengue incidence increased consecutively in the pre-dengue season months that year were extracted to explore decision-making as a response to rising cases. Finally, for the ‘outbreak management’ scenario, only provinces with outbreaks exceeding a set outbreak threshold for 3 months during this period were selected.

For each operational scenario, a probabilistic accuracy assessment was conducted, based on the exceedance probabilities of the 4 outbreak thresholds provided within the D-MOSS forecasting system. Based on an endemic channel (the number of dengue cases per month and per province over the previous 5 years), the thresholds include the endemic channel plus one standard deviation, endemic channel plus two standard deviations, and both the 75^th^ percentile and 95^th^ percentile of the distribution of dengue cases per month [17].

For each province, the observed values were categorised into binary variables based on whether the dengue cases exceeded each threshold (1) or not (0), and the probability of the outbreak threshold being exceeded was extracted from the forecasted data, using an arithmetic mean across the 42 seasonal climate forecast ensembles.

Probabilistic classification assessments were performed for each outbreak scenario, each outbreak threshold and both decision-making opportunity month forecasts (April and May). A range of accuracy metrics were calculated for each scenario and outbreak threshold to evaluate utility performance. pROC v1.18.5 [35] in R was used to generated receiver operating characteristic curves (ROC)- a diagnostic curve plotting the true positive rate against the false positive rate)- which the area under the ROC curve (AUC) value summarises into a single value between 0 and 1. This value represents the probability D-MOSS will correctly distinguish a randomly chosen exceedance event and a randomly chosen non-exceedance event. For each scenario and outbreak threshold, the Brier Score was generated, by calculating the mean squared difference between predicted exceedance probabilities and the observed binary outcome of whether the threshold was exceeded or not, to quantify probabilistic accuracy.


$$Brier\;Score=\;\frac{\text{1}}{N}\sum_{n=\text{1}}^{N}{\left(f_{n}-\;y_{n}\right)}^{\text{2}}$$

where $f_{n}$ are predicted probabilities that the outbreak will be exceeded ($y_{n}$ = 1). Across the continuous range of probabilities, other metrics were calculated, including accuracy (the proportion of correct predictions),


$$Accuracy=\;\frac{True\;Positives+True\;Negatives}{True\;Positives+True\;Negatives+False\;Positives+False\;Negatives}$$

sensitivity (the probability that observed positive instances were forecasted),


$$Sensitivity=\frac{True\;Positives}{True\;Positives+False\;Negatives}$$

and positive predictive value (the probability that forecasted positive instances were observed).


$$Positive\;Predictive\;Value\;(PPV)=\frac{True\;Positives}{True\;Positives+False\;Positives}$$

Binary classification assessments for discrete probability thresholds (0.25, 0.5 and 0.75) were contextualised in proportional contingency bar plots with decision-making classifications.

## Results and discussion

### Dengue dynamics during operational period

In total, there were 692,617 observed dengue cases throughout the operational period (July 2019- September 2022) across all 63 provinces. These exhibited notable spatial distributions, with higher numbers of dengue cases seen in southern and central provinces (424,402 and 158,404 respectively), compared to highland (60,211) and northern provinces (47,600). High population areas reported higher number of cases; Ho Chi Minh city province reported 17% of overall cases (120,161), with the bordering Dong Nai and Binh Duong provinces, and Hanoi in the north, all reporting over 30,000 cases each to be the four highest provinces. When population-normalised to an incidence rate per 100,000 people, the highest incidence rates were still seen in southern and central provinces, however the cities no longer reported the highest incidence rates. Such spatial distributions have been reported previously, with endemic dengue dynamics in the south and more sporadic outbreaks in the north, restricted mostly to Ha Noi and the Red River Delta [26].

**Fig 1:**
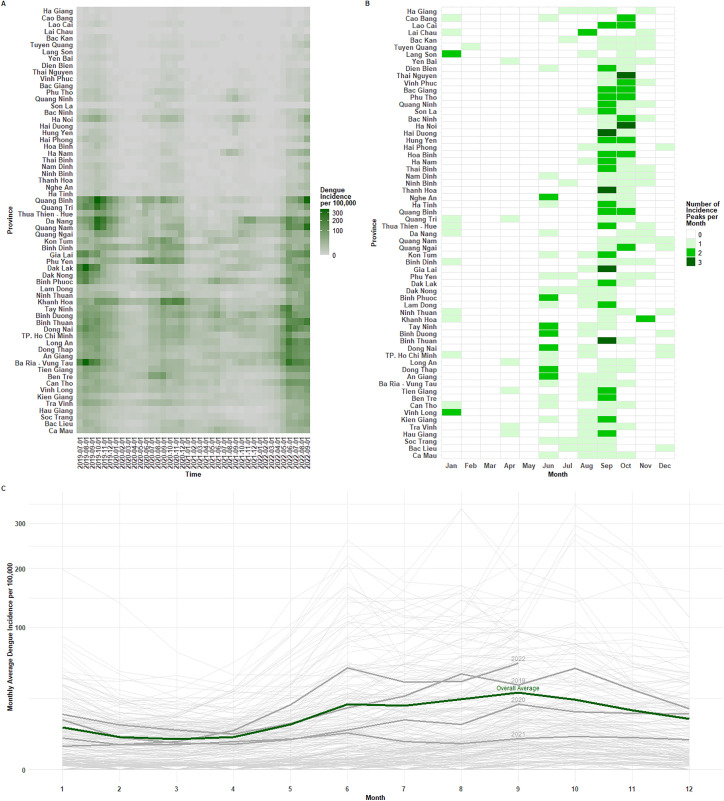
Distribution of key dengue outcomes during operational period; A) Heatmap of dengue incidence per 100,000 people across provinces and time; B) Heatmap of count of instances dengue cases peaked in each month across provinces; B) Line graph of seasonality of dengue cases in each province, with annual and overall arithmetic means calculated across provinces and years respectively.

Temporally, dengue incidence was higher in the second half of the year (Fig 1a) with 41.7% of cases reported between August and October. In terms of the months when cases peak in each province each year during the operational period, one of the outcome metrics, (Fig 1b) peaks were observed in September across all regions (n = 83), and then October (n = 56), fitting dengue seasonality patterns previously observed in Vietnam [26], with September of 2022 and October of 2019 being particularly notable peak months across many provinces.

Alongside this seasonality, the operational period exhibited interannual variability (Fig 1c), with higher interannual variability identified among northern regions (coefficient of variation 1.15) than southern regions (coefficient of variation 0.83). Generally, lower incidence was seen in 2020 and 2021, with 2022 reporting the highest number of cases (despite being an incomplete year, with the operational period ending at the end of September). This year recorded the two highest number of cases per month- June 2022 (63,220) and September 2022 (58,404), followed by October 2019, August 2019 and July 2022.

Notably, 2022 saw an early rise in observed dengue cases in June, with the 31 peaks observed in June found in this year, mostly associated with northern provinces. The timing and magnitude of this early seasonal peak was uncharacteristic for Vietnam. It has previously been suggested that this early peak may have been due to a resurgence of transmission after the relaxation of COVID-19 related restrictions that suppressed dengue transmission and led to accumulation of human susceptibility [36,37]. While fluctuations in host immunity are common during natural dengue epidemic cycles [38], with 10–12 years usually between epidemics in Vietnam, Covid-19 measures that suppressed dengue transmission (such as reduced human mobility or time spent in high-risk transmission areas) could have accelerated the increase of susceptibility, shortening this epidemic cycle [37].

It is important to note that the operational period only contains two full years (2020 and 2021) of data, with only partial coverage of the calendar year for the higher-incidence years of 2019 and 2022. However, this period remains representative of dengue dynamics in Vietnam. For example, the annual dengue case counts for 2019–2022 fitted within a similar annual range and peaked at similar timing to previous analyses by Gibb et al., [26] from 1998-2021.

### Global performance metrics across dimensions

Global performance metrics calculated across all D-MOSS Vietnam dengue forecasts (including all seasonal climate forecast ensembles and lead times) over the operational period provided insights of overall performance (Table 2). D-MOSS dengue forecasts outperformed the random predictor and seasonal expanding average baseline in almost all metrics representative of dengue dynamics, highlighting areas where the D-MOSS forecast system adds value (in terms of reducing errors and generating more accurate predictions).

**Table 2:** Overall performance metrics of D-MOSS forecast compared to random baseline and seasonal expanding average baseline forecast performance metrics. Three selected metrics represent dengue dynamics: point incidence accuracy is measured by root mean squared error (RMSE), peak timing difference is measured by temporal difference between peaks (in months), and trajectory shape accuracy is measured by a distance metric calculated by dynamic time warping as the minimal cumulative cost required to align the observed and forecasted time series of dengue cases. Arithmetic mean of each metric was generated across all months and provinces for each lead time and averaged across all lead times. Areas where value has been added by D-MOSS forecast (error reductions from baseline approaches) are shaded.

Performance Metric	D-MOSS Forecasts	Seasonal Baseline	Difference	Random Baseline	Difference
**RMSE (overall)**	**25.70**	**31.29**	**−5.59**	**40.95**	**−15.25**
RMSE (lead time 1)	20.35	23.93	−3.59	42.20	−21.86
RMSE (lead time 2)	26.02	28.28	−2.27	41.33	−15.32
RMSE (lead time 3)	27.96	32.55	−4.59	41.50	−13.54
RMSE (lead time 4)	28.52	33.14	−4.63	40.48	−11.97
RMSE (lead time 5)	25.37	34.43	−9.05	39.96	−14.58
RMSE (lead time 6)	25.99	35.38	−9.39	40.20	−14.21
**Peak Difference (overall)**	**2.55**	**4.09**	**−1.54**	**4.52**	**−1.97**
Peak Difference (lead time 1)	2.08	2.38	−0.30	4.08	−2.00
Peak Difference (lead time 2)	2.27	3.14	−0.87	4.27	−2.00
Peak Difference (lead time 3)	2.44	3.85	−1.40	4.27	−1.83
Peak Difference (lead time 4)	2.61	4.41	−1.80	4.66	−2.05
Peak Difference (lead time 5)	2.79	5.00	−2.21	4.81	−2.02
Peak Difference (lead time 6)	3.11	5.76	−2.65	5.04	−1.93
**Trajectory Distance (overall)**	**310.63**	**310.63**	**−0.01**	**527.50**	**−216.87**
Trajectory Distance (lead time 1)	248.36	265.23	−16.87	592.07	−343.71
Trajectory Distance (lead time 2)	306.33	268.46	37.87	564.89	−258.56
Trajectory Distance (lead time 3)	326.12	308.16	17.96	546.32	−220.20
Trajectory Distance (lead time 4)	341.90	312.60	29.30	504.76	−162.87
Trajectory Distance (lead time 5)	329.05	341.99	−12.94	484.79	−155.74
Trajectory Distance (lead time 6)	312.01	367.37	−55.36	472.15	−160.14

D-MOSS had a notably lower root mean squared error score, suggesting performance strength in point predictions, with a 37.2% and 17.8% improvement over the province-specific random predictor and seasonal expanding average baseline respectively overall across all provinces, months and lead times. Similar improvements were noted for predictions of dengue peak timing, with both the random and seasonal expanding average baseline averaging at a temporal difference of over 4 months between predicted and observed peak month, however the D-MOSS overall average of a 2.5 month temporal difference required further analysis into regions where peaks are more difficult to predict. While D-MOSS reported higher performance at predicting trajectories over the random baseline (with a 41.2% improvement), there was fewer differences between the seasonal expanding average baseline and the D-MOSS trajectory distances overall (across all lead times), however evaluating the performance metrics across each lead time of the D-MOSS forecast horizon (n = 1–6) revealed more specific insights into how D-MOSS performance varies across the forecast horizon.

**Fig 2:**
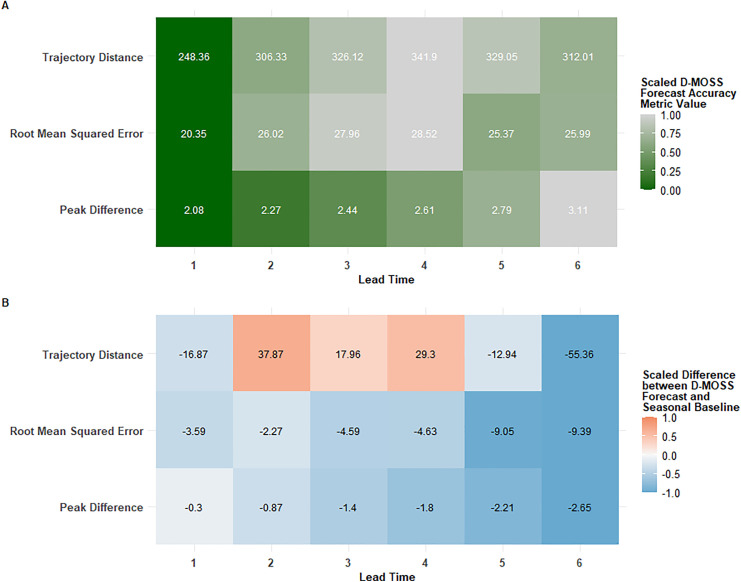
D-OSS forecast performance metrics across lead times, with comparison to a seasonal expanding average baseline. Metrics representative of dengue outcomes used are mean squared error in incidence, temporal distance between observed and forecasted peak of incidence (months), and trajectory distance based on dynamic time warping analysis. A) Heatmap of metrics averaged across lead times; B) Heatmap of metric differences between the D-MOSS forecast and the seasonal expanding average baseline averaged across lead times. Colour bars have been scaled to facilitate comparative visualisation of metrics with distinct magnitudes: accuracy metric values are scaled between 0-1, with darker shaded values indicating higher errors; difference values are scaled between -1 and 1, with a true 0, with negative numbers indicating improvements provided by D-MOSS (blue), and positive numbers highlighting areas when D-MOSS reported higher errors than a seasonal baseline (red).

Value was added over a seasonal expanding average baseline, in terms of reducing errors and improving predictions, for almost all metrics across all lead times, with the exception of trajectory forecast errors at medium-term 2–4 month lead times. The performance of shorter-term lead times of 1 month exceeded those of all other lead times by a large margin (Fig 2). However, this was not always followed by a linear decline in subsequent longer-term lead times, expected based on historical accuracy assessments in Colon-Gonzalez et al. [17], with only the peak difference metric displaying this behaviour. Intuitively, performance would be expected to decrease as lead time increases, due to dengue forecasting difficulty increasing as the horizon stretches into more distant months, however lead times of 3 and 4 months were found to have the highest RMSE and least value-added compared to the baseline models.

The value added, in terms of differences between the D-MOSS forecasts and baseline models, occasionally exhibited the opposite behaviour- such as the improvements in RMSE over the seasonal expanding average baseline, which increased with longer-lead times (up to a 26.5% improvement). Additionally, we found that the value-added by D-MOSS for forecasting peak timing of dengue cases increased with longer-term lead times, possibly suggesting the system is able to predict when the seasonal pattern is likely to deviate, and the subsequent peak will occur differently from the previous years. This suggests that D-MOSS longer forecast horizons can, unlike seasonal models, adapt to factors that might drive mid-range seasonal shifts, up to a 6 month timeframe, highlighting the value of the inclusion of seasonal climate forecasts which become important contributors to D-MOSS forecasts in later lead times. This is particularly pertinent amidst the novel use of seasonal climate forecasts for prospective dengue forecasting within D-MOSS in an operational setting, highlighting their value in generating longer-term predictions.

### Temporal

**Fig 3:**
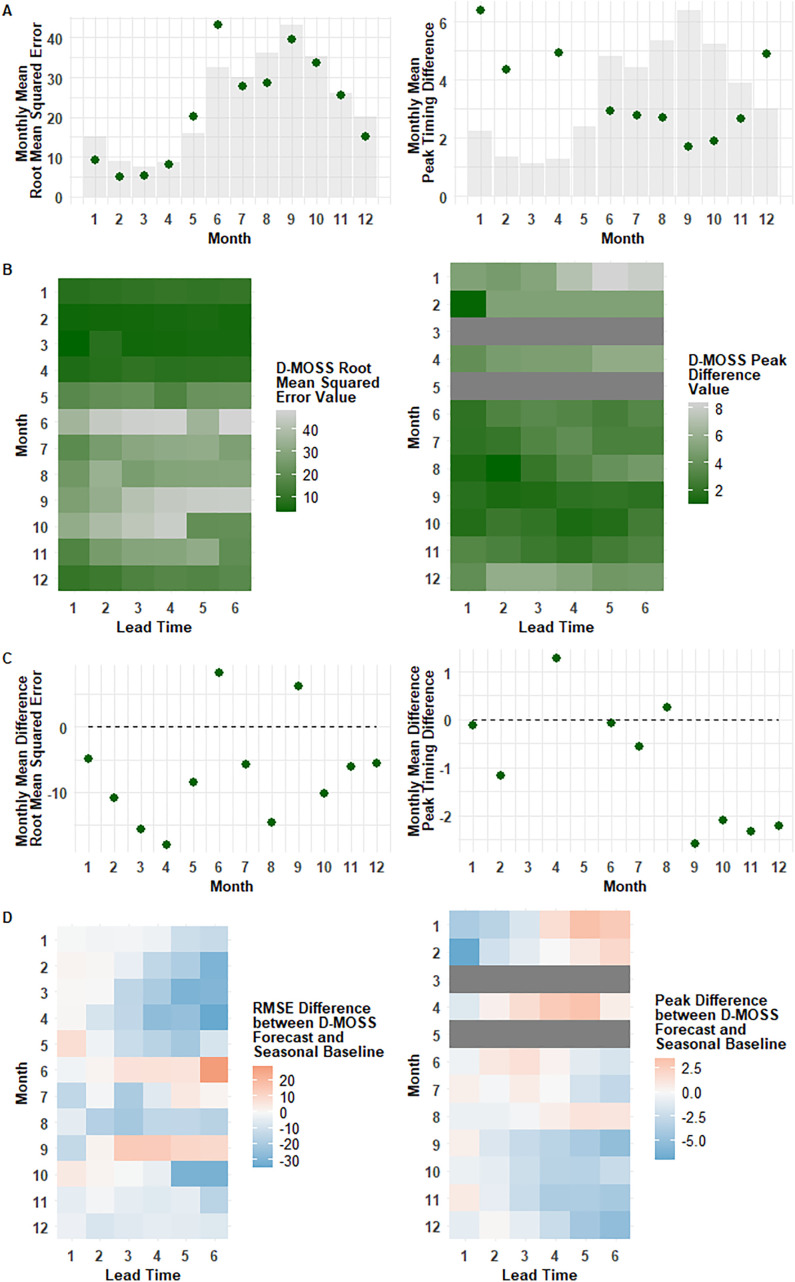
Temporal patterns of monthly D-MOSS forecast performance across lead times, with comparison to a baseline seasonal expanding average. The dengue season in Vietnam usually falls between June and October. Monthly performance was evaluated for root mean squared error (RMSE) in incidence (left-hand plots) and temporal distance between months when peaks of incidence were observed and forecasted (right-hand plots). A) Overall monthly metric means represented by dots over scaled bar plots of dengue incidence per metric for visual reference to seasonal dengue variation; B) Heatmap of monthly metric means across lead times; C) Overall monthly metric mean differences between the D-MOSS forecast and the seasonal expanding average baseline represented by dots, with the dashed line at 0 difference highlighting values below 0 that indicate superior D-MOSS performance; D) Heatmap of monthly mean metric differences between the D-MOSS forecast and the seasonal expanding average baseline across lead times, with negative numbers indicating improvements provided by D-MOSS (blue), and positive numbers highlighting occasions when D-MOSS reported larger errors than a seasonal baseline (red). No peaks were reported in March or May in any province, so these rows have been shaded out in grey.

Performance was found to vary seasonally when the representative metrics were calculated for each month, and across lead times (Fig 3). This temporal variation could only be calculated for mean squared error and the peak timing, as the trajectory distance is calculated based on the time series as a whole rather than specific monthly dengue forecasts. In terms of RMSE of predicted dengue incidence, fewer errors were found in the early months of the year, when dengue incidence is lowest (Fig 3a), with the largest errors found in June (ranging from 35.5 to 48.2 across all lead times). Dengue forecasts in September exhibited a linear drop-off in accuracy as the lead time increases (Fig 3b), however this pattern was less pronounced in other months, with a notable non-linear relationship found for October dengue forecasts where longer lead times had lower errors, suggesting the long-term forecast for October (made in April or May, before the dengue season begins) is more accurate for this month than mid-season forecasts.

These D-MOSS dengue forecasts added value over a seasonal expanding average baseline (Fig 3c), in terms of reducing mean squared error, for most months. For some months, such as October to December, this value was added at all lead times (Fig 3d), but at other times of the year, most notably for June and September, D-MOSS dengue forecasts reported higher error rates than the seasonal expanding average baseline at longer lead times, particularly 3–6 months (Fig 3d), resulting in a higher overall RMSE for these months (Fig 3c). There was therefore an opposing performance trend for dengue forecasts in high incidence months, as they reported both the highest accuracy (August and October), and the lowest accuracy (June and September), however in the latter case, early lead times still added value over a seasonal baseline (reducing RMSE by 1.8 and 13.2 respectively for lead times of 1 month).

In terms of forecasting the month in which dengue cases would peak, D-MOSS was able to predict peaks more accurately in the second half of the year (Fig 3a) when the majority of peaks were recorded (Fig 1b), particularly for a one month lead time when the error distance averaged at 1.4 to 1.9 months between June and October (Fig 3b), but with the largest improvements over the seasonal expanding average baseline often reported for longer lead times later in the year (Fig 3d), highlighting strong predictive potential of dengue forecasts issued at the start of the dengue season in June and July. Poorer peak prediction accuracy is expected in the earlier half of the year when there are minimal numbers of peaks, mostly in northern provinces with low incidence where the classification of ‘peaks’ become more ambiguous. Overall, the D-MOSS dengue forecasts had higher errors predicting out-of-season peaks, but did add value compared to the seasonal expanding average baseline in the second half of the year, particularly at longer lead times, likely as the climate variables included in the seasonal climate forecasts have a stronger influence on transmission during the usual dengue season [37] to drive peak behaviour.

Months when D-MOSS added less value for the magnitude of dengue cases (incidence RMSE), such as September, reported greater value added in the prediction of peaks, suggesting the forecast can be useful each month for different purposes, driving the rationale for evaluating performance holistically over a range of outcomes.

The impact of the uncharacteristically early June 2022 outbreaks, as mentioned previously, becomes clear in the poorer performance observed in June for both performance metrics (Fig 3). The D-MOSS dengue forecasts did not capture this high magnitude outbreak in advance through longer lead time forecasts issued earlier in the year, with the peak only forecasted correctly in one province at a lead time of one month (S2 Fig). The D-MOSS forecasting system itself does not currently capture the specific drivers that have been suggested as responsible for this outbreak- such as population immunity dynamics linked to Covid-19 restrictions being relaxed [37] or the statistically significant serotype shift reported in some regions in 2022, such as in Quang Nam where DENV-1 became the most prevalent serotype, rising from 5.95% of positive samples in 2020–2021 to 51.32% in 2022 [39]. Conversely, the predominant dengue serotype in southern Vietnam shifted in 2022 to DENV-2 [40]. Such temporal analysis highlights a specific example of when climactic factors, that are currently being used for the predictions, contribute less to dengue dynamics amidst confounding drivers of virus serotype dynamics. Variations in seasonality driven by external factors such as government interventions, human movement and population immunity pose challenges for D-MOSS dengue forecasts, which would require data to represent these confounding factors, such as from seroprevalence surveys or routine proportional serotype data, to more accurately forecast this specific outbreak.

### Spatial

**Fig 4:**
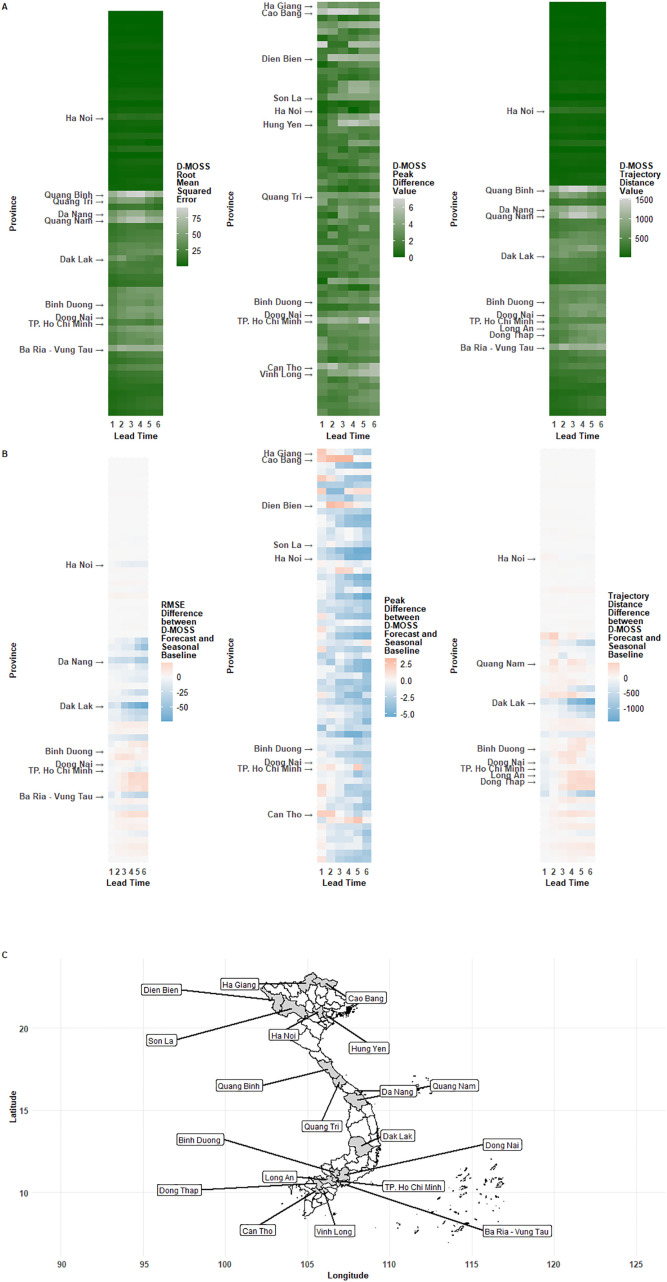
Spatial patterns of D-MOSS forecast performance across provinces and lead times, with comparison to a baseline seasonal expanding average. From left to right, plots correspond to the following performance metrics: root mean squared error (RMSE) in incidence, temporal distance between months of observed and forecasted peak of incidence, and trajectory distance based on dynamic time warping analysis. A) Heatmap of province-specific metric means across lead times. B) Heatmap of province-specific mean metric differences between the D-MOSS forecast and the seasonal expanding average baseline across lead times, with negative numbers indicating improvements provided by D-MOSS (blue), and positive numbers highlighting occasions when D-MOSS reported larger errors than a seasonal baseline (red). In heatmaps, provinces are ordered by declining latitude from top to bottom, and provinces that reported highest number of dengue cases (Ho Chi Minh, Dong Nai, Binh Duong and Ha Noi) and results-specific provinces are labelled. C) Locations of all labelled provinces. Administrative area shapefiles provided by Global Administrative Areas database (https://gadm.org/download_country.html).

Exploration into province-specific accuracy metrics revealed spatial variation in D-MOSS performance (Fig 4, S3 Fig). Larger RMSE values were found in central provinces such as Quang Binh, Quang Tri, Da Nang and Quang Nam (Fig 4a), and southern provinces such as Bà Rịa–Vũng Tàu, while northern and the southernmost provinces had the most accurate incidence forecasts.

A similar pattern was exhibited for trajectory errors (Fig 4a), however the temporal distance between forecasted peak months exhibited less pronounced spatial patterns apart from a distinct clustering of errors in northern provinces, such as Ha Giang and Cao Bang, that exhibit more ambiguous dengue seasonality to define peaks (Fig 4a). Very few provinces displayed linear drop-off trends in accuracy as lead times increased, with variability between provinces larger than variability between lead times within-province. Of the provinces that reported the most dengue cases in the operational period (Ho Chi Minh, Dong Nai, Binh Duong and Ha Noi- highlighted in Fig 4), high accuracy across all metrics was found for Ha Noi, across most metrics for Ho Chi Minh (apart from trajectory distance), whereas Dong Nai and Binh Duong had relatively high error rates compared to other provinces. Both of these provinces border Ho Chi Minh province with notable human movement patterns, and this metropolitan expansion results in greater peri-urban environments including sub-urban villages and agricultural land.

The overall trends in spatial performance suggested the model adds less value compared to baseline models in southern provinces, where dengue is epidemic and incidence remains consistently high, for RMSE and trajectory distance metrics (Fig 4b), however this value was skewed by poorer performance of later lead times- in many cases, early lead times of 1 month still added value in these provinces (such as Long An and Dong Thap for trajectory distance). This suggests local health authorities in these provinces could effectively use D-MOSS as a short-term planning tool, in combination with leveraging real-time entomological surveillance signals, in a combined approach for early warning- still enabling enable timely vector control campaigns and targeted public health messaging.

Particular value is added by D-MOSS (in terms of reducing mean squared error compared to the seasonal baseline) across all lead times in provinces such as Da Nang, Dak Lak and Bà Rịa–Vũng Tàu, despite sometimes having high error rates. This suggests dengue dynamics are likely harder to predict in these provinces which could be associated with variable human mobility to these areas- with Da Nang a major urban centre, Bà Rịa–Vũng Tàu a popular coastal tourist area, and internal migration to agricultural areas in Dak Lak- with the D-MOSS model able to capture these more complex dynamics better than a seasonal baseline.

There were no clear trends of trajectory accuracy difference across lead times in each province (Fig 4b), apart from a clustering of southern provinces (particularly in Long An, Dong Thap and An Giang) when the difference between D-MOSS forecasted trajectories outperformed the seasonal expanding average trajectories at a lead time of 1 month, but underperformed in later lead times. Similarly, mid-lead times added less value in provinces such as Quang Nam in central Vietnam. D-MOSS added predictive power over a seasonal expanding average forecasted trajectory particularly in one province, Dak Lak, increasing into the longer lead times similar to the RMSE values.

Unlike the RMSE and trajectory results, value was added in almost every province overall for peak forecasting (Fig 4b). Notably, longer-lead times reported the highest improvements over seasonal baselines, with early and mid-range lead times adding the least value over the seasonal expanding average baseline. Lead times where less value were being added tended to be in the north, (e.g., in northern provinces like Ha Giang and Cao Bang) where outbreaks are more sporadic [26].

### Operational utility for decision-making

**Fig 5:**
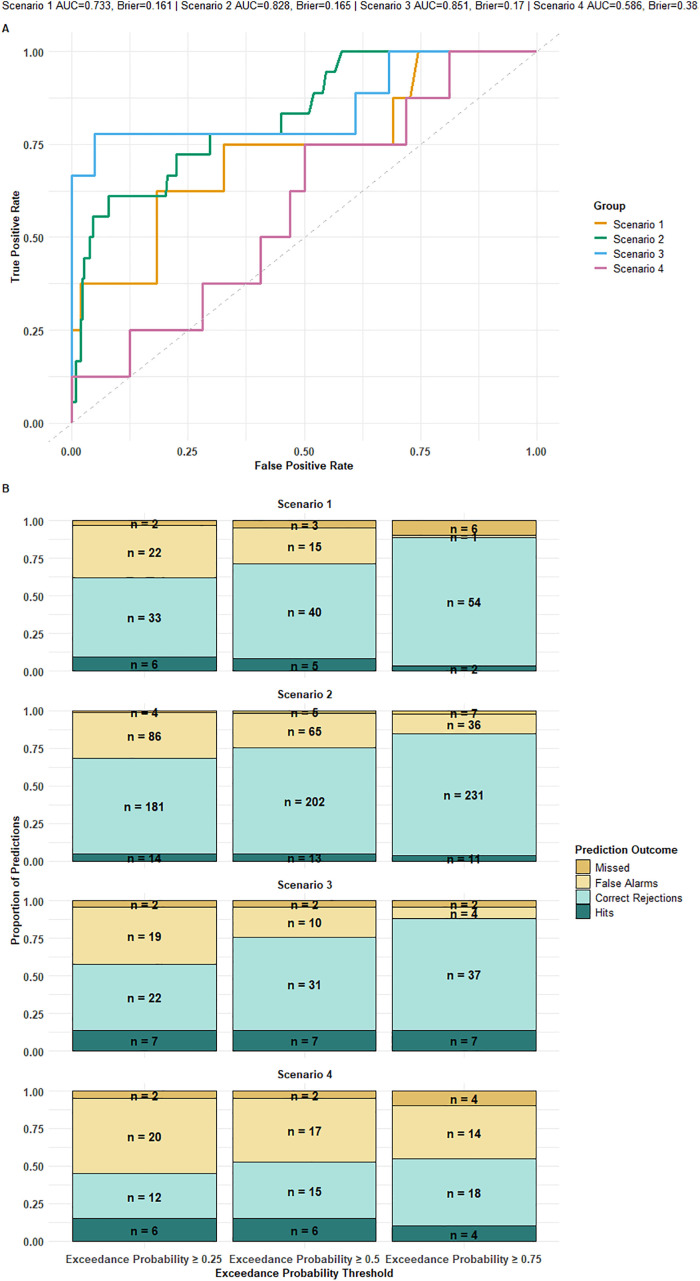
D-MOSS utility performance assessment based on probabilistic classification of four operational dengue scenarios; budget allocation (scenario 1), forecasting (scenario 2), early warning (scenario 3), and outbreak management (scenario 4), described fully in in Table 1. These results are based on forecasts issued in April (focusing on the forecast horizon June to October to be comparable with May forecasts), and use the ‘mean plus two standard deviations’ outbreak threshold as one of the four thresholds available within the D-MOSS user interface. (A) Receiver operating characteristic (ROC) curve for each operational scenario. (B) Bar plots contextualising proportion of hits (true positives, dark blue), correct rejections (light blue), false alarms (light yellow) and missed outbreak exceedances (dark yellow) for three discrete probability cut-offs (0.25, 0.5 and 0.75), for each operational scenario.

The accuracy of the D-MOSS utility assessment relevant to decision-making varied across the selected operational scenarios tested using the mean plus two standard deviation outbreak threshold in 2020 (Fig 5). The AUC scores (Fig 5a) ranged from 0.586 to 0.851, and Brier scores from 0.161-0.383 across all operational scenarios for the first decision-making opportunity month (April in this context). The highest AUC was reported for scenario 3 (early warning), while the lowest, and therefore best, Brier score was reported for scenario 1 (budgeting), with scenarios 1–3 reporting below 0.2. Based on Hosmer Jr et al. [41], an AUC score below 0.7 (as seen only for scenario 4, outbreak management) can be considered poor whereas the other scenarios that were higher could be considered acceptable (scenario 1) or excellent in the case of scenarios 2 and 3 which exceeded 0.8.

Accuracy metrics were calculated for each scenario across the full range of probability cutoffs (0–1, S4 Fig). Generally, the highest accuracy for all operational scenarios (0.825 to 0.94) was reported when the probability of exceeding the outbreak threshold was higher (0.71 to 0.98), however the imbalanced inputs (in which there were more instances of non-exceedances) likely drives this relationship, as higher probability cut-offs would conservatively classify most samples as negative, and the high proportion of true negatives would disproportionately improve accuracy- hence the range of alternative metrics assessed alongside accuracy. Generally, PPV increased and sensitivity decreased as the probability threshold was set higher, as expected due to more conservative probabilities reducing the rate of false positives, but in turn decreasing the detection rate of actual positives. Scenario 3 exhibited a consistently high sensitivity which only dropped off around a probability of 0.8, compared to the more steady decline observed for other scenarios, offering a possible optimum probability cut-off for that operational scenario.

Contextualised contingency tables (Fig 5b) for discrete exceedance probability cut-offs (0.25, 0.5 and 0.75) revealed patterns of binary classifications of outbreak exceedance. Higher probabilities of exceedance resulted in fewer false alarms for all operational scenarios, with only 1 false alarm reported for scenario 1 at a 0.75 probability cut-off. For scenarios 1, 2 and 4, false negatives (missed outbreak threshold exceedances) increased as the probability cut-off increased. The number of ‘hits’ and ‘missed’ outbreak exceedance events remained the same for scenario 3 for all probability cut-offs. In terms of use, lower probability cut-offs could be beneficial for budgeting and resource allocation, with the acceptance of potential false positives to ensure preparedness, however higher probability cut-offs would be more appropriate for outbreak response and targeted interventions, to minimize false alarms and prioritize actions where confidence is highest.

Facilitating comparison to other forecasting systems, the accuracy metrics for each operational scenario for this outbreak threshold were calculated for a 0.5 probability cut-off. Sensitivity, which is important to reduce the number of missed opportunities to act in the decision-making process, ranged from 0.722 to 0.778 for scenarios 2–4, with a lower sensitivity reported for scenario 1 (0.625). This lies within a similar range to those calculated for dengue forecasting systems previously of 57–97% [23,24,42] despite the fact that these models were either predicting outbreaks at only 2–3 months in advance, compared to the D-MOSS forecast horizon up to 6 months tested here, or optimally calibrated for specific municipalities unlike the D-MOSS national implementation. While these results were promising, there was a high false alarm rate observed at the 0.5 probability cut-off with low PPV values between 0.166 and 0.412. These results were lower than those previously reported for EWARS dengue forecasting systems [20,21,23,24]. Conversely, accuracy was high, ranging from 0.714 to 0.782 for scenarios 1–3, due to a strong specificity in predicting true negatives, with a lower value for scenario 4 (0.525).

Spatially, based on this 0.5 probability cut-off, the false alarms across scenarios 1 and 2 particularly, were clustered in southern provinces (S5 Fig). Northern and central highlands provinces were generally correctly predicted, with the exception of Son La in the north-west, which was classified as a missed outbreak exceedance (false negative) in scenarios 1, 3 and 4. Another province, Kon Tum, was also classified as a false negative in multiple scenarios, suggesting D-MOSS dengue forecasts under-estimated the dengue dynamics of that year in that province, which would have resulted in missed opportunities to use interventions proactively.

The accuracy of information in the first decision-making opportunity month compared to waiting for the next month’s forecast (May in this context) produced diverging results (S6 Fig). While the AUC values increased for all operational scenarios in the May forecast slightly (ranging from 0.016 to 0.055 increases), with the highest reported increase for the previously lowest-performing scenario (scenario 4), the Brier scores also increased, indicating poorer probabilistic accuracy (S6 Fig). The improvements observed mostly consisted of reduced false negatives at the 0.75 probability cut-off, including two reduced false negatives for scenario 1 (and no false alarms) and one less false negative for scenario 4. In the context of decision-making, early action could be implemented based on the first decision-making opportunity month, and then additional provinces could be mobilised the next month as the updated forecast identifies new true positives (that were previously missed). However, there were increased false alarms for scenario 2 and 3 at all probability cut-offs, suggesting the issues driving false alarms were not fixed by waiting for the next decision-making opportunity a month later. This suggests, for some scenarios, the forecasts for both months could be interpreted in combination, however the marginal increase in accuracy identified by waiting for the next month’s forecast, suggests acting sooner using the longer-term forecast horizon was mostly as informative.

The operational scenario utility accuracy differs based on the user selection of the four outbreak exceedance thresholds offered within the D-MOSS system. The more conservative 75th percentile outbreak threshold had more balanced input datasets, with a greater proportion of outbreak exceedances, resulting in a higher number of correct hits, less false alarms but more false negatives particularly at higher probability cut-offs (S7 Fig). This threshold therefore had lower sensitivity values, but higher PPV values such as 0.86 for scenario 1 and 0.63 for scenario 2 for the 0.5 probability cut-off. The 95th percentile outbreak threshold (S8 Fig) and the mean plus one standard deviation outbreak threshold (S9 Fig) had less false alarms also. The 95th percentile outbreak threshold had lower AUC values for all scenarios other than outbreak management, due to low sensitivity to outbreak exceedances at higher probability cut-offs. The mean plus one standard deviation outbreak threshold had lower Brier scores than the mean plus two standard deviation outbreak threshold, suggesting this threshold could be better calibrated in terms of probabilities, but not as effective as distinguishing exceedances from non-exceedances.

Spatially, compared to the mean plus two standard deviation (S3 Fig), the other outbreak thresholds false alarms in the southernmost provinces, particularly for scenario 1 but with notable reductions in Scenario 2 using the 95th percentile threshold (S10 Fig). For the 75th and 95th percentile outbreak thresholds there were increased false negatives in northern provinces (S10 Fig); in low incidence areas the distribution of dengue cases is highly skewed by frequent low numbers of cases and occasional ‘spikes’, which can result in disproportionately high percentile outbreak thresholds which can lead to a low detection rate.

These diverging results suggest D-MOSS forecasts perform heterogeneously based on both the outbreak threshold selected and across different regions, suggesting local decision-makers should choose the appropriate outbreak threshold for local dengue dynamics and response capabilities, or based on the prioritisation of particular accuracy metrics. Generally, the accuracy of the utility assessment appears lower than expected based on the holistic global performance metrics, with these results highlighting two main limitations of D-MOSS forecasts through the poor performance observed for scenario 4 (outbreak management) and the general trend of false alarms decreasing the PPV. However this is likely the result of the inflexible classification approach. Imposing strict threshold boundaries can oversimplify complex dengue dynamics, penalising small errors disproportionately when the forecast was almost correct but just marginally below an arbitrary threshold. This is particularly true of scenario 4; while most of the operational scenarios offered valuable forecasting information for decision-makers, scenario 4 reported particularly low AUC, PPV and accuracy values. However the target for this classification, in identifying the single month with the highest exceedance threshold probability to estimate when an outbreak might peak, is a very narrow target compared to general outbreak detection, with a more imbalanced classification set-up than the other scenarios, with near-misses (such as a one month discrepancy) being penalised, and difficulty increasing in southern provinces when all months might exhibit high exceedance probabilities to delineate the single peak.

Actionable public health interventions often require forecasts to have a reasonably high predictive confidence (such as AUC ≥ 0.8), suggesting D-MOSS forecasts are currently most suitable for direct decision-making for the early warning and forecasting scenario, where the AUC exceeded this threshold. Incorrect predictions might still be informative in a utility sense (for example early warning of an increase that has been marginally overpredicted is still useful when impacts could be severe), particularly when D-MOSS is used for decision-making alongside entomological or virus sequence data which can inform more accurate responses around marginal predictions. In this way, for scenarios with lower AUC (annual budgeting and outbreak management), D-MOSS outputs can still be valuable for guiding heightened surveillance or public risk communication, while direct operational decisions may require additional corroborating data sources.

### Limitations

Prospective dengue forecasting using real time data in an operational setting presents particular challenges, such as reporting delays which can reduce predictive accuracy [43]. In Vietnam, an average dengue reporting delay of 10 days has been documented [44], but the use of confirmed rather than suspected cases results in more finalised data being received by D-MOSS. Another operational issue are interruptions to this data pipeline [45], such as for the satellite-derived co-variates, however the D-MOSS system approach of allowing for missing days of data each month appears robust in dealing with such interruptions. Unusual interruptions to the dengue cases data pipeline were reported in many regions during 2020–2022 due to delays in laboratory confirmations of infections as resources were reallocated towards the pandemic effort [46]. This operational period in general presented unique challenges for dengue forecasting- ranging from an unusually large outbreak year in 2019 resulting in high immunity levels [36], to two years of reduced transmission amidst pandemic-related complexities, an early unprecedented dengue season in 2022, and various immunity fluxes throughout. Additional influences on the 2020 operational utility assessments include government interventions that reduced mobility [47], resulting in an estimated reduced incidence of 34% from April to December 2020 in Vietnam [36], or concerns of under-reporting due to reduced treatment-seeking and availability or laboratory testing [48] associated with the Covid-19 pandemic, particularly for Vietnam’s hospital-based dengue surveillance system. However, in reality, there was little evidence for the latter with no recorded increase in fatality rate proportions which are usually associated with a skew in reporting only severe cases [36]. Therefore, while 2020 remains a representative year, with 138,693 reported dengue cases similar to the average 130,235 dengue cases a year between 2013–2019 [13,14], it is possible pandemic-related reduction in incidence could have increased false positives in the utility assessments; where behavioural and mobility changes negated the usual climate and sociodemographic variables driving the D-MOSS dengue forecasting system.

Additionally, we could not account for the effect of any resulting dengue control activities that were initiated based on the D-MOSS forecast in our analyses. While D-MOSS was included as a tool for dengue control in the updated national guidelines since November 2020, its usage by local decision-makers is not yet systematically recorded. Discussions from project workshops indicate anecdotally that D-MOSS is currently used mostly for reactive interventions for the next month, suggesting any likely impact on the evaluation would be largely limited to the 1-month lead-time forecasts and the outbreak management utility scenario.

A further challenge is evaluating a national forecasting system across provinces with widely varying dengue incidence. Although incidence rates were used to normalise for population differences to account for magnitude discrepancies, metrics such as RMSE and trajectory distance still reflected this magnitude disparity, with lower-incidence areas reporting less errors and smaller ranges of value differences. Comparisons with systems like EWARS become difficult as these are designed specifically for high-priority areas, whereas D-MOSS currently prioritises operation across a heterogeneous national landscape. Despite this variability, performance ranges across provinces were relatively narrow, highlighting how D-MOSS performs effectively across such heterogeneous epidemiological settings.

It is also difficult to compare directly with previous retrospective accuracy assessments of the D-MOSS forecasting system on historical data, due to minor model adjustments made pre-deployment, however these results provide initial insights into the efficacy to effectiveness gap discussed previously. A notable difference was the positive performance of later lead times (4–6 months); whereas previously the 4–6 month lead times were found to be less accurate than a baseline model [17], highlighting the effective inclusion of seasonal climate forecasts for prospective dengue forecasting.

### Areas of future development

The results of both the global performance metrics and the utility assessment highlight particular areas of future development that should be prioritised for D-MOSS in Vietnam, and wider dengue forecasting systems. Including longer-term immunity levels (found to affect both the 2020 utility assessments and the poor performance for the uncharacteristic June 2022 peak), human movement data and seroprevalence data would facilitate capturing fluctuations driving dengue dynamics not currently considered within the D-MOSS dengue forecasting system. The latter two had been suggested previously [36], with shifting predominant dengue serotype previously used as a predictor of outbreak risk to sensitivities of 50–99% and PPVs of 71–80% in Malaysia [20], but this indicator is not currently available across the entirety of Vietnam. Future inclusion of longer-term lagged dengue cases could offer a crude variable to explain some of this interannual variation. While additional datasets could address some specific challenges, prospective forecasting will always face unprecedented events affecting disease dynamics (discussed in [37]). Developing an inverse modelling approach, using backward-time inference to reconstruct latent epidemiological or environmental conditions behind unexpected dengue peaks (such as June 2022), could improve forecast calibration and robustness, but at the trade-off of higher computational cost, increased interpretation difficulty for decision-makers and potential overfitting to specific outbreak patterns.

Increasing the spatial resolution of D-MOSS dengue forecasts from a province to a district level would facilitate inclusion oflocal-level urban infrastructure and human mobility that can predict local spatial patterns of dengue incidence [26], particularly beneficial in central and southern provinces with higher incidence and higher error rates, and urban provinces to delineate peri-urban dynamics and microhabitats that drive *Aedes* populations in Vietnam [26,49]. In the context of utility, dengue interventions are manged locally, supporting the need for finer-scale forecasts for locality-level decision-making. This was confirmed in a user acceptance testing study of D-MOSS in Malaysia [50], alongside similar recommendations for districts to be the focal geographical unit from users of EWARS dengue forecasting tools [20].

Future development should improve user-defined prioritised accuracy metrics. For example, costly interventions might prioritise PPV to reduce false alarms, while high-severity decisions might prioritise minimizing false negatives for timely opportunity to mitigate severe consequences. Lead-time accuracy can also be prioritised according to decision-making timelines. Locally, D-MOSS forecasts need to be adapted by selecting appropriate outbreak thresholds or probability cut-offs for local-level dynamics, and accuracy assessments should be guided by user needs to ensure relevance for operational decisions.

While the operational period here offered unique opportunities for performance evaluation of how complex dengue dynamics are captured within the D-MOSS forecasting system, a longer operational period would facilitate the comparison of utility accuracy assessments in high incidence years with numerous outbreaks, compared to lower incidence years; or a comparison of ‘business-as-usual’ years compared to the unprecedented conditions associated with 2019–2022.

## Conclusions

This evaluation has summarised how D-MOSS dengue forecasts perform across a range of spatiotemporal dimensions and lead times, highlighting implications for operational use in terms of where and when the forecast is accurately capturing dengue dynamics for confident decisions, or areas when the model is currently informative but could be improved to increase operational utility. Users have previously highlighted a strength of the D-MOSS forecasting system is its ability to forecast up to six months in advance to facilitate proactive policy development including coordination of dengue prevention and control activities. This analysis now confirms that these longer-term forecasts retain their accuracy in real-world conditions, promoting the continued use of forecasts in this way and suggesting opportunities to forecast further into the future. Certain areas where D-MOSS can be refined for effective dengue management have been identified, including increasing spatial resolution to improve prediction of local dengue dynamics, characterising non-climactic influences on dengue dynamics as seen during the operational period (such as longer-term immunity and human mobility), and ensuring the forecasts are used most effectively (in terms of appropriate outbreak thresholds and probability cut-offs for both particular vector control interventions or local dengue dynamics).

While this evaluation has been performed specifically for Vietnam, the holistic approach and challenges identified are applicable to any operational dengue forecasting system. Recommendations for future operational evaluations of such forecasting systems include focusing on a range of performance metrics rather than just point assessments, such as the dengue trajectory and peak timing analysis here, as these alternative forecasted dynamics offer unique performance information to users. Additionally, assessments should be multidimensional, exploring interactions between forecast horizons, space and time as performed here, rather than just reporting overall metrics, to facilitate more focused direction for downstream dengue forecasting system improvements. The impact of the D-MOSS forecasting system operationally has been strengthened by the system being easy to and understand, able to run on various browsers and devices, and not requiring users to have specialized statistical knowledge. Evaluations should similarly be developed with users in mind, specifically developing utility scenarios with the forecast users to provide more contextualised performance assessment alongside statistical accuracy, to promote forecasting integration into dengue management protocols.

## Supporting information

S1 Fig Schematic showing monthly data used for operational utility analysis for each scenario, in terms of D-MOSS issued forecasts, forecast horizons tested, and monthly data used to select provinces based on selection criteria outlined in Table 1. (DOCX)

S2 Fig Provinces where a dengue incidence peak was observed in June 2022 (A), compared to the province-level D-MOSS forecast for peaks (B). The only correctly predicted June 2022 peak was in Tay Ninh. Administrative area shapefiles provided by Global Administrative Areas database (https://gadm.org/download_country.html).
(DOCX)

S3 Fig Choropleth maps of spatiotemporal patterns of D-MOSS performance metrics and value added by forecast compared to a baseline seasonal expanding average. The first column corresponds to the mean squared error in incidence, the second to temporal distance between observed and forecasted peak of incidence (months), and the third to trajectory distance based on dynamic time warping analysis. a) Choropleth map of overall province metric averages; b) Choropleth map of overall province metric average differences between the D-MOSS forecast and the seasonal expanding average baseline. Administrative area shapefiles provided by Global Administrative Areas database (https://gadm.org/download_country.html).
(DOCX)

S4 Fig D-MOSS utility performance assessment line plots of sensitivity and positive predictive values (PPV) alongside accuracy as a continuous function of probability of outbreak threshold (mean plus two standard deviations) being exceeded for each operational scenario; budget allocation (scenario 1), forecasting (scenario 2), early warning (scenario 3), and outbreak management (scenario 4), described fully in in Table 1. (DOCX)

S5 Fig Spatial trends in utility assessment accuracy across provinces, based on a 50% probability of exceeding the mean plus two standard deviations outbreak threshold for (A) budget allocation scenario, (B) forecasting scenario, (C) early warning scenario and (D) outbreak management scenario. Administrative area shapefiles provided by Global Administrative Areas database (https://gadm.org/download_country.html).
(DOCX)

S6 Fig D-MOSS utility performance assessment results for second decision-making opportunity month (April) based on probabilistic classification of four operational dengue scenarios; budget allocation (scenario 1), forecasting (scenario 2), early warning (scenario 3), and outbreak management (scenario 4), described fully in in Table 1. These results are based on forecasts issued in May (focusing on the forecast horizon June to October to be comparable with April forecasts), and use the ‘mean plus two standard deviations’ outbreak threshold as one of the four thresholds available within the D-MOSS user interface. (A) Receiver operating characteristic (ROC) curve for each operational scenario. (B) Bar plots contextualising proportion of hits (true positives, dark blue), correct rejections (light blue), false alarms (light yellow) and missed outbreak exceedances (dark yellow) for three discrete probability thresholds (0.25, 0.5 and 0.75), for each operational scenario.
(DOCX)

S7 Fig D-MOSS utility performance assessment for 75^th^ percentile outbreak threshold based on probabilistic classification of four operational dengue scenarios; budget allocation (scenario 1), forecasting (scenario 2), early warning (scenario 3), and outbreak management (scenario 4), described fully in in Table 1. These results are based on forecasts issued in April (focusing on the forecast horizon June to October to be comparable with May forecasts), and use the ‘75^th^ percentile’ outbreak threshold as one of the four thresholds available within the D-MOSS user interface. (A) Receiver operating characteristic (ROC) curve for each operational scenario. (B) Bar plots contextualising proportion of hits (true positives, dark blue), correct rejections (light blue), false alarms (light yellow) and missed outbreak exceedances (dark yellow) for three discrete probability thresholds (0.25, 0.5 and 0.75), for each operational scenario.
(DOCX)

S8 Fig D-MOSS utility performance assessment for 95th percentile outbreak threshold based on probabilistic classification of four operational dengue scenarios; budget allocation (scenario 1), forecasting (scenario 2), early warning (scenario 3), and outbreak management (scenario 4), described fully in in Table 1. These results are based on forecasts issued in April (focusing on the forecast horizon June to October to be comparable with May forecasts), and use the ‘95^th^ percentile’ outbreak threshold as one of the four thresholds available within the D-MOSS user interface. (A) Receiver operating characteristic (ROC) curve for each operational scenario. (B) Bar plots contextualising proportion of hits (true positives, dark blue), correct rejections (light blue), false alarms (light yellow) and missed outbreak exceedances (dark yellow) for three discrete probability thresholds (0.25, 0.5 and 0.75), for each operational scenario.
(DOCX)

S9 Fig D-MOSS utility performance assessment for the mean plus one standard deviation outbreak threshold based on probabilistic classification of four operational dengue scenarios; budget allocation (scenario 1), forecasting (scenario 2), early warning (scenario 3), and outbreak management (scenario 4), described fully in in Table 1. These results are based on forecasts issued in April (focusing on the forecast horizon June to October to be comparable with May forecasts), and use the ‘75^th^ percentile’ outbreak threshold as one of the four thresholds available within the D-MOSS user interface. (A) Receiver operating characteristic (ROC) curve for each operational scenario. (B) Bar plots contextualising proportion of hits (true positives, dark blue), correct rejections (light blue), false alarms (light yellow) and missed outbreak exceedances (dark yellow) for three discrete probability thresholds (0.25, 0.5 and 0.75), for each operational scenario.
(DOCX)

S10 Fig Spatial trends in utility assessment accuracy across provinces, based on a 50% probability of exceeding the outbreak thresholds for budget allocation scenario, forecasting scenario, early warning scenario and outbreak management scenario. (A) 75^th^ percentile outbreak threshold, (B) 95^th^ percentile outbreak threshold, (C) mean plus one standard deviation outbreak threshold. Administrative area shapefiles provided by Global Administrative Areas database (https://gadm.org/download_country.html).
(DOCX)
