# Supplementary material for: Performance evaluation of an operational dengue forecasting system (D-MOSS) in Vietnam
Source: PLOS Glob Public Health. 2026 Mar 6;6(3):e0005867. doi: 10.1371/journal.pgph.0005867 (PMC12965583; doi:10.1371/journal.pgph.0005867)

**S1 Fig: Schematic showing monthly data used for operational utility analysis for each scenario,** in terms of D-MOSS issued forecasts, forecast horizons tested, and monthly data used to select provinces based on selection criteria outlined in Table 1.


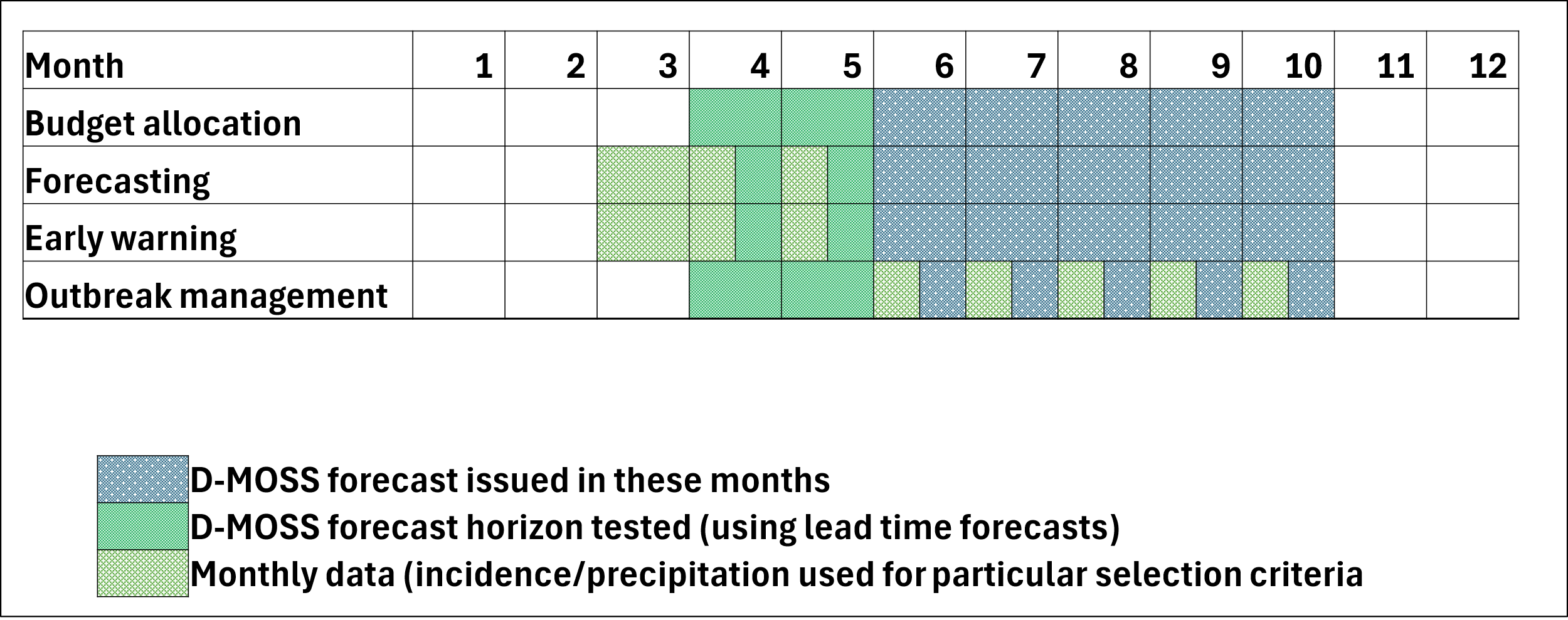

Supplement: S1 Fig — (DOCX) [file pgph.0005867.s001.docx]
