# Supplementary material for: Performance evaluation of an operational dengue forecasting system (D-MOSS) in Vietnam
Source: PLOS Glob Public Health. 2026 Mar 6;6(3):e0005867. doi: 10.1371/journal.pgph.0005867 (PMC12965583; doi:10.1371/journal.pgph.0005867)

**S2 Fig: Provinces where a dengue incidence peak was observed in June 2022 (A), compared to the province-level D-MOSS forecast for peaks (B)**. The only correctly predicted June 2022 peak was in Tay Ninh. Administrative area shapefiles provided by Global Administrative Areas database (https://gadm.org/download_country.html).


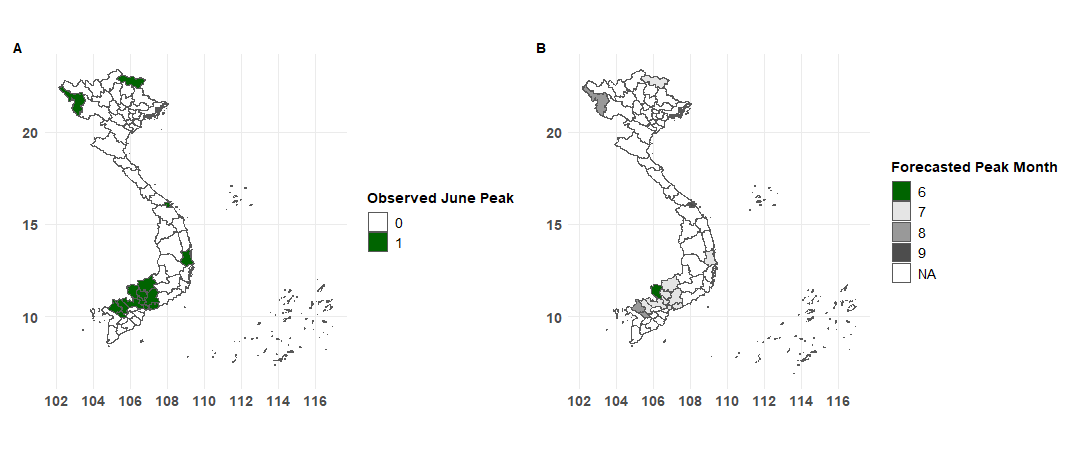

Supplement: S2 Fig — The only correctly predicted June 2022 peak was in Tay Ninh. Administrative area shapefiles provided by Global Administrative Areas database (https://gadm.org/download_country.html). (DOCX) [file pgph.0005867.s002.docx]
