# Supplementary material for: Performance evaluation of an operational dengue forecasting system (D-MOSS) in Vietnam
Source: PLOS Glob Public Health. 2026 Mar 6;6(3):e0005867. doi: 10.1371/journal.pgph.0005867 (PMC12965583; doi:10.1371/journal.pgph.0005867)
Supplement: S3 Fig — The first column corresponds to the mean squared error in incidence, the second to temporal distance between observed and forecasted peak of incidence (months), and the third to trajectory distance based on dynamic time warping analysis. a) Choropleth map of overall province metric averages; b) Choropleth map of overall province metric average differences between the D-MOSS forecast and the seasonal expanding average baseline. Administrative area shapefiles provided by Global Administrative Areas database (https://gadm.org/download_country.html). (DOCX) [file pgph.0005867.s003.docx]

**S3 Fig: Choropleth maps of spatiotemporal patterns of D-MOSS performance metrics and value added by forecast compared to a baseline seasonal expanding average.** The first column corresponds to the mean squared error in incidence, the second to temporal distance between observed and forecasted peak of incidence (months), and the third to trajectory distance based on dynamic time warping analysis. a) Choropleth map of overall province metric averages; b) Choropleth map of overall province metric average differences between the D-MOSS forecast and the seasonal expanding average baseline. Administrative area shapefiles provided by Global Administrative Areas database (https://gadm.org/download_country.html).

**
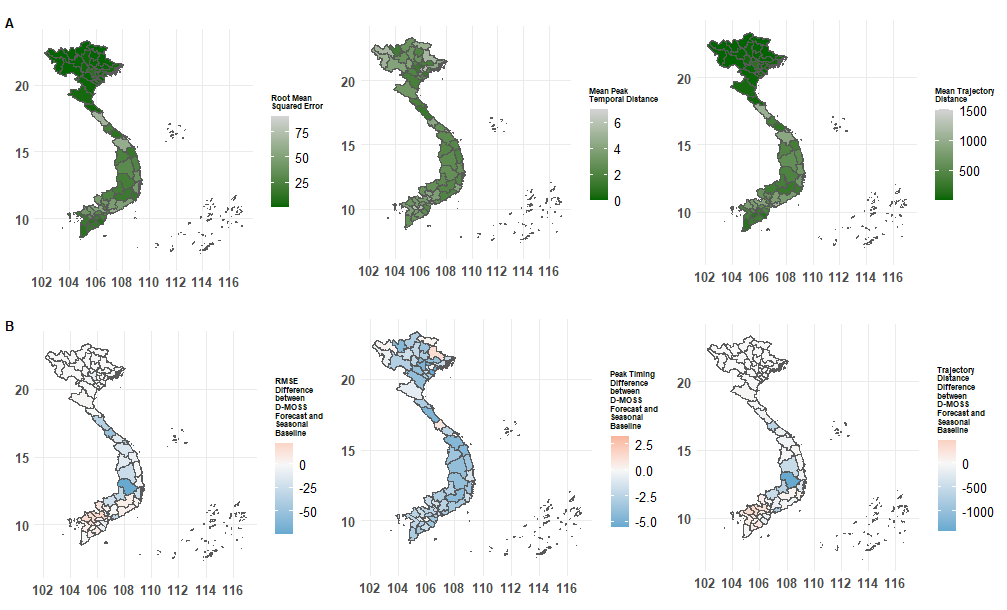
**
