# Supplementary material for: Performance evaluation of an operational dengue forecasting system (D-MOSS) in Vietnam
Source: PLOS Glob Public Health. 2026 Mar 6;6(3):e0005867. doi: 10.1371/journal.pgph.0005867 (PMC12965583; doi:10.1371/journal.pgph.0005867)
Supplement: S4 Fig — (DOCX) [file pgph.0005867.s004.docx]

**S4 Fig: D-MOSS utility performance assessment line plots of sensitivity and positive predictive values (PPV) alongside accuracy as a continuous function of probability of outbreak threshold (mean plus two standard deviations) being exceeded for each operational scenario**; budget allocation (scenario 1), forecasting (scenario 2), early warning (scenario 3), and outbreak management (scenario 4), described fully in in Table 1.

**
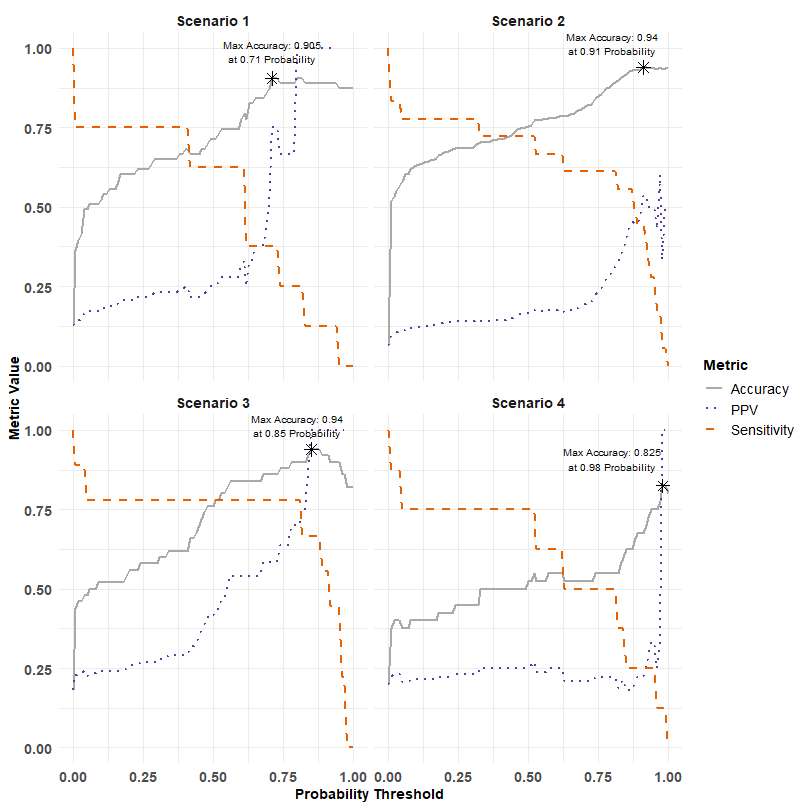
**
