# Supplementary material for: Performance evaluation of an operational dengue forecasting system (D-MOSS) in Vietnam
Source: PLOS Glob Public Health. 2026 Mar 6;6(3):e0005867. doi: 10.1371/journal.pgph.0005867 (PMC12965583; doi:10.1371/journal.pgph.0005867)
Supplement: S5 Fig — Administrative area shapefiles provided by Global Administrative Areas database (https://gadm.org/download_country.html). (DOCX) [file pgph.0005867.s005.docx]

**S5 Fig: Spatial trends in utility assessment accuracy across provinces, based on a 50% probability of exceeding the mean plus two standard deviations outbreak threshold for (A) budget allocation scenario, (B) forecasting scenario, (C) early warning scenario and (D) outbreak management scenario.** Administrative area shapefiles provided by Global Administrative Areas database (https://gadm.org/download_country.html).

**
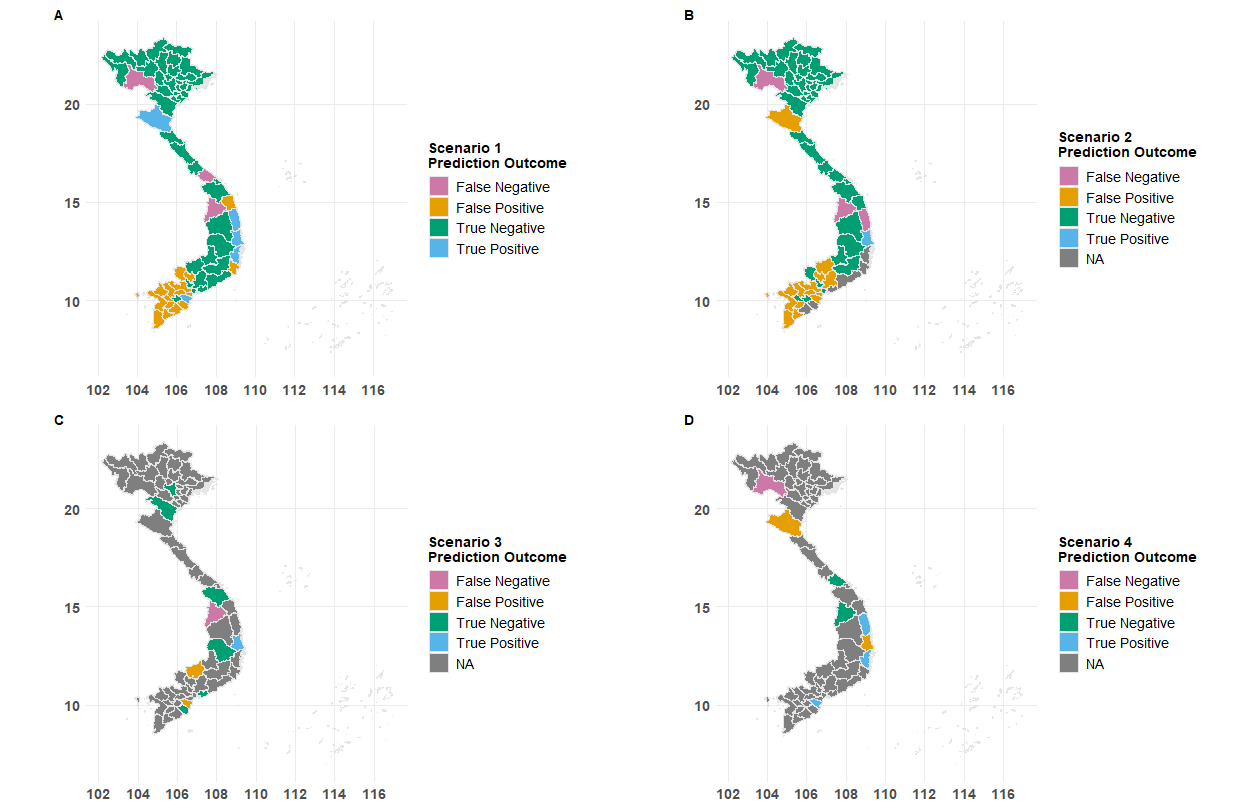
**
