# Supplementary material for: Performance evaluation of an operational dengue forecasting system (D-MOSS) in Vietnam
Source: PLOS Glob Public Health. 2026 Mar 6;6(3):e0005867. doi: 10.1371/journal.pgph.0005867 (PMC12965583; doi:10.1371/journal.pgph.0005867)
Supplement: S9 Fig — (A) Receiver operating characteristic (ROC) curve for each operational scenario. (B) Bar plots contextualising proportion of hits (true positives, dark blue), correct rejections (light blue), false alarms (light yellow) and missed outbreak exceedances (dark yellow) for three discrete probability thresholds (0.25, 0.5 and 0.75), for each operational scenario. (DOCX) [file pgph.0005867.s009.docx]

**S9 Figure: D-MOSS utility performance assessment for the mean plus one standard deviation outbreak threshold based on probabilistic classification of four operational dengue scenarios**; budget allocation (scenario 1), forecasting (scenario 2), early warning (scenario 3), and outbreak management (scenario 4), described fully in in Table 1. These results are based on forecasts issued in April (focusing on the forecast horizon June to October to be comparable with May forecasts), and use the ‘75^th^ percentile’ outbreak threshold as one of the four thresholds available within the D-MOSS user interface. (A) Receiver operating characteristic (ROC) curve for each operational scenario. (B) Bar plots contextualising proportion of hits (true positives, dark blue), correct rejections (light blue), false alarms (light yellow) and missed outbreak exceedances (dark yellow) for three discrete probability thresholds (0.25, 0.5 and 0.75), for each operational scenario.

**
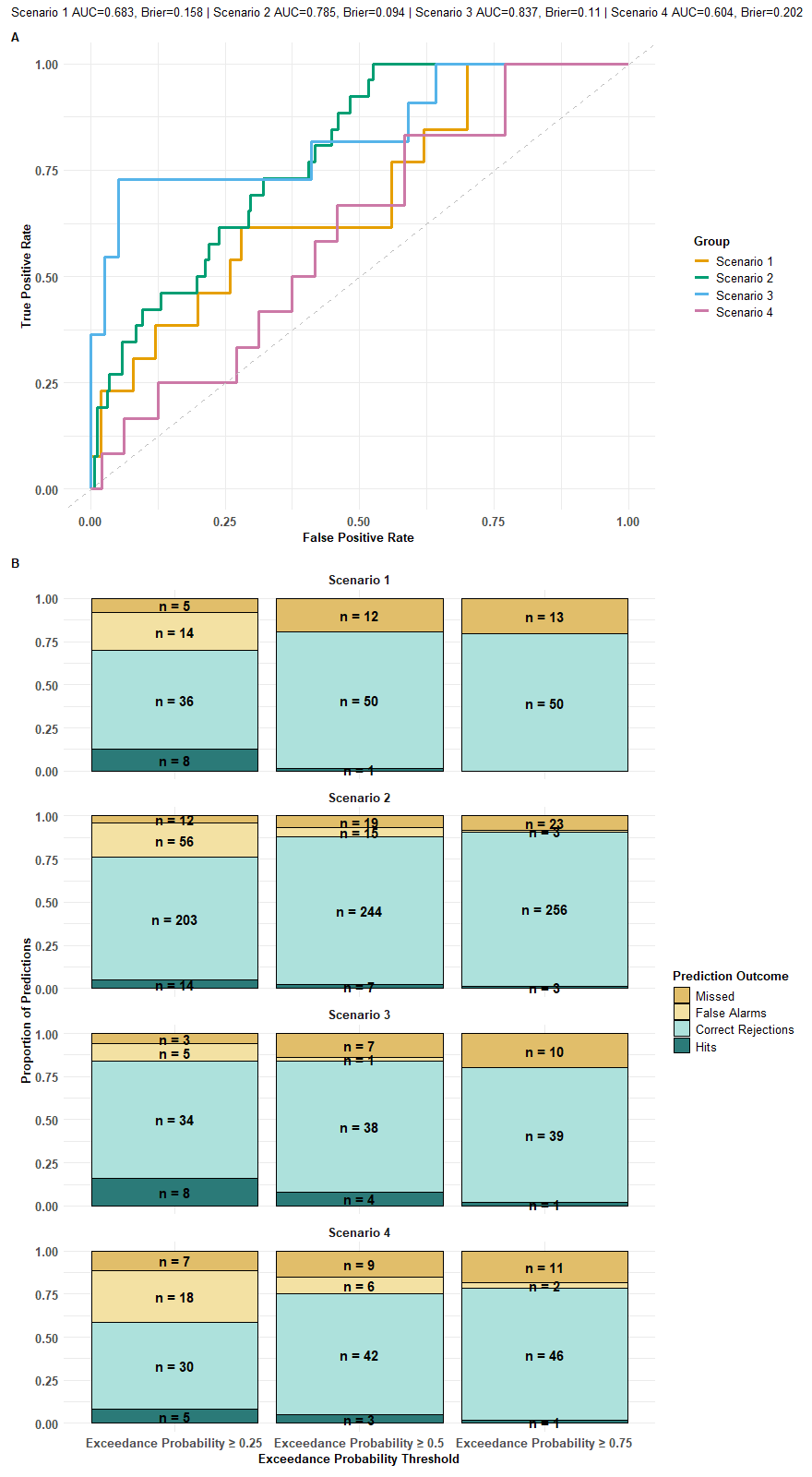
**
