# Supplementary material for: Performance evaluation of an operational dengue forecasting system (D-MOSS) in Vietnam
Source: PLOS Glob Public Health. 2026 Mar 6;6(3):e0005867. doi: 10.1371/journal.pgph.0005867 (PMC12965583; doi:10.1371/journal.pgph.0005867)
Supplement: S10 Fig — (A) 75th percentile outbreak threshold, (B) 95th percentile outbreak threshold, (C) mean plus one standard deviation outbreak threshold. Administrative area shapefiles provided by Global Administrative Areas database (https://gadm.org/download_country.html). (DOCX) [file pgph.0005867.s010.docx]

**S10 Fig: Spatial trends in utility assessment accuracy across provinces, based on a 50% probability of exceeding** **the outbreak thresholds** for budget allocation scenario, forecasting scenario, early warning scenario and outbreak management scenario. (A) 75^th^ percentile outbreak threshold, (B) 95^th^ percentile outbreak threshold, (C) mean plus one standard deviation outbreak threshold. Administrative area shapefiles provided by Global Administrative Areas database (https://gadm.org/download_country.html).

**
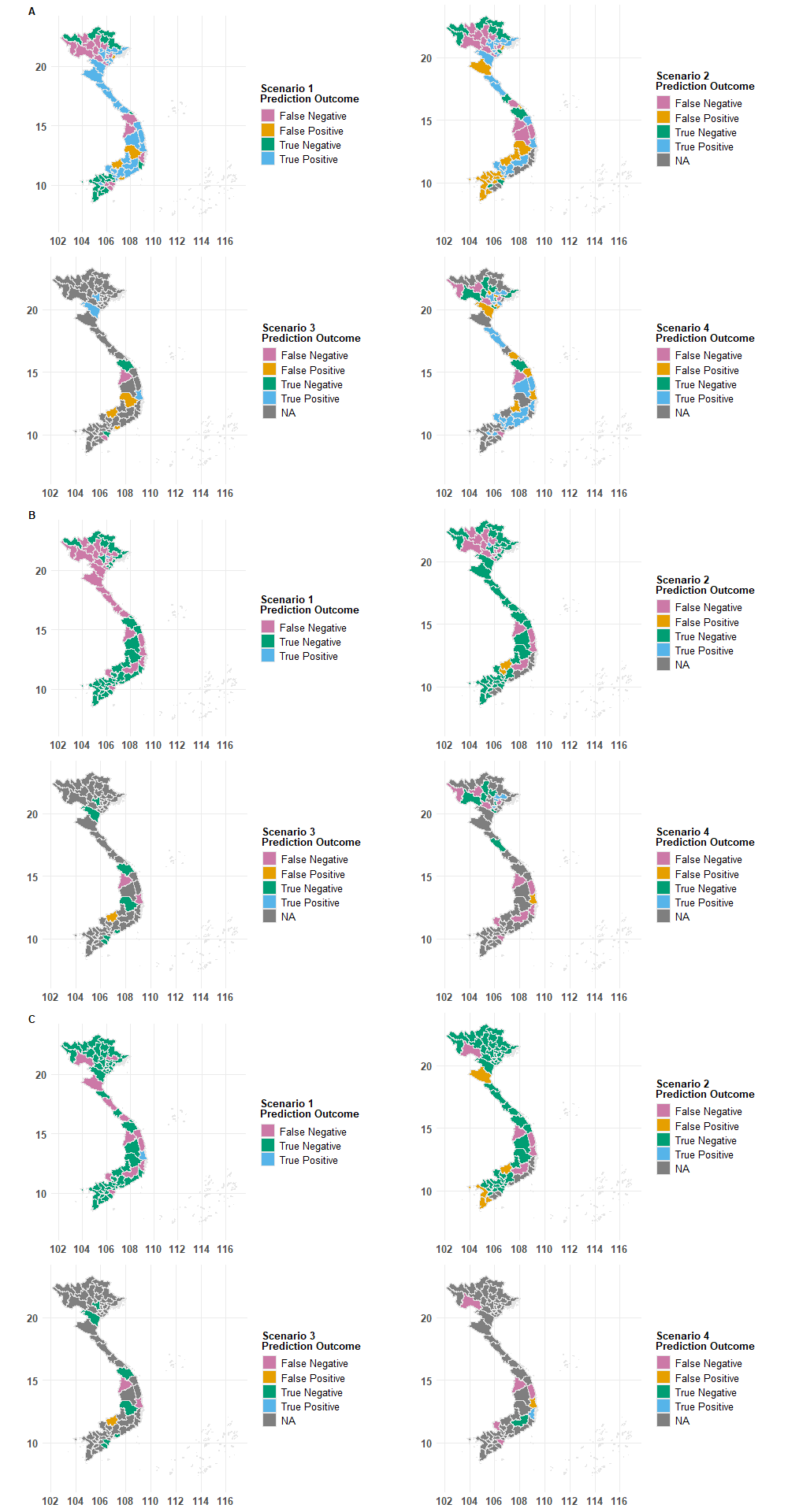
**
